# Low‐Temperature Reverse Water–Gas Shift Enabled by Magnetically Induced Catalysis

**DOI:** 10.1002/anie.202523576

**Published:** 2026-01-28

**Authors:** Junhui Hu, Lise Marie Lacroix, Jacob Johny, Sourav Ghosh, Elisabeth Hannah Wolf, Jeongmin Ji, Sheng‐Hsiang Lin, Manisha Durai, Alin Benice Schöne, Walid Hetaba, Holger Ruland, Walter Leitner, Alexis Bordet

**Affiliations:** ^1^ Max Planck Institute For Chemical Energy Conversion Mülheim an der Ruhr Germany; ^2^ Institut Für Technische und Makromolekulare Chemie RWTH Aachen University Aachen Germany; ^3^ Laboratoire De Physique et Chimie des Nano‐Objets Université De Toulouse, LPCNO, INSA, UPS CNRS‐UMR 5215 Toulouse France; ^4^ Institut Universitaire De France (IUF) Paris France; ^5^ Department of Materials Science and Engineering Gwangju Institute of Science and Technology (GIST) Gwangju Republic of Korea

**Keywords:** Cu/Al2O3, Fe@C nanoparticles, magnetically induced catalysis (MICat), reverse water gas shift, thermodynamic equilibrium

## Abstract

Equilibrium‐limited endothermic reactions play a crucial role in the transition toward a more sustainable chemical industry, but are typically plagued by the need for high operation temperatures (>500°C). Here, we show that the temperature gradients generated by the selective and localized heating of catalyst materials in a colder reactor environment shift the equilibrium of thermodynamically‐limited endothermic reactions and improve their performance. In particular, the reverse water gas shift reaction and magnetic induction are selected as the model reaction and selective catalyst heating method, respectively. Magnetically induced catalysis using standard Cu–Al spinel‐derived catalyst functionalized with carbon‐coated iron nanoparticles enables high CO yield (up to 62%) at mild catalyst and reactor temperatures (estimated at 300°C and determined as 25–123°C, respectively). We demonstrate that the catalyst temperature and not the reactor temperature governs the equilibrium product composition of the rWGS, and that the temperature gradient promotes the in situ removal of water to shift the gas phase thermodynamic equilibrium. These two points synergistically result in a CO yield that would require a reactor temperature of 650°C in a conventionally heated gas phase reaction.

## Graphical abstract

Magnetically induced catalysis using standard Cu–Al spinel‐derived catalyst functionalized with carbon‐coated iron nanoparticles enables high CO yield (up to 62%) at mild catalyst and reactor temperatures (estimated at 300°C and determined as 25–123°C, respectively). The catalyst temperature and not the reactor temperature govern the equilibrium product composition of the rWGS, and the temperature gradient promotes the in situ removal of water to shift the gas phase thermodynamic equilibrium.

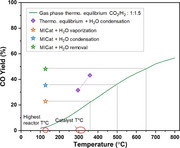

## 1 Introduction

Equilibrium‐limited endothermic reactions are crucial for the chemical industry and pivotal to the establishment of a sustainable energy‐chemistry nexus. Prominent examples include the production of CO from CO_2_ via the reverse water gas shift (rWGS) reaction [1, 2], the production of syngas via steam methane reforming [3, 4], and the generation of hydrogen from the decomposition of methanol [5] and ammonia [6]. However, these gas phase reactions must be operated at high temperatures (>500°C) to shift the equilibrium sufficiently for delivering sufficient productivity. These extreme temperatures in the reaction chamber are typically achieved by heating the reactor walls employing mostly conventional burners fired with fossil fuels, which poses severe challenges with regard to investment costs, energy consumption, and efficiency, and reactor and catalyst degradation [1, 2, 3, 4, 5, 6, 7, 8, 9]. The development of efficient and cost‐effective catalytic technologies for such reactions is essential to meet the growing demand for energy carriers and chemicals while reducing the environmental impact of industrial chemical processes and mitigating climate change [7].

**FIGURE 1:**
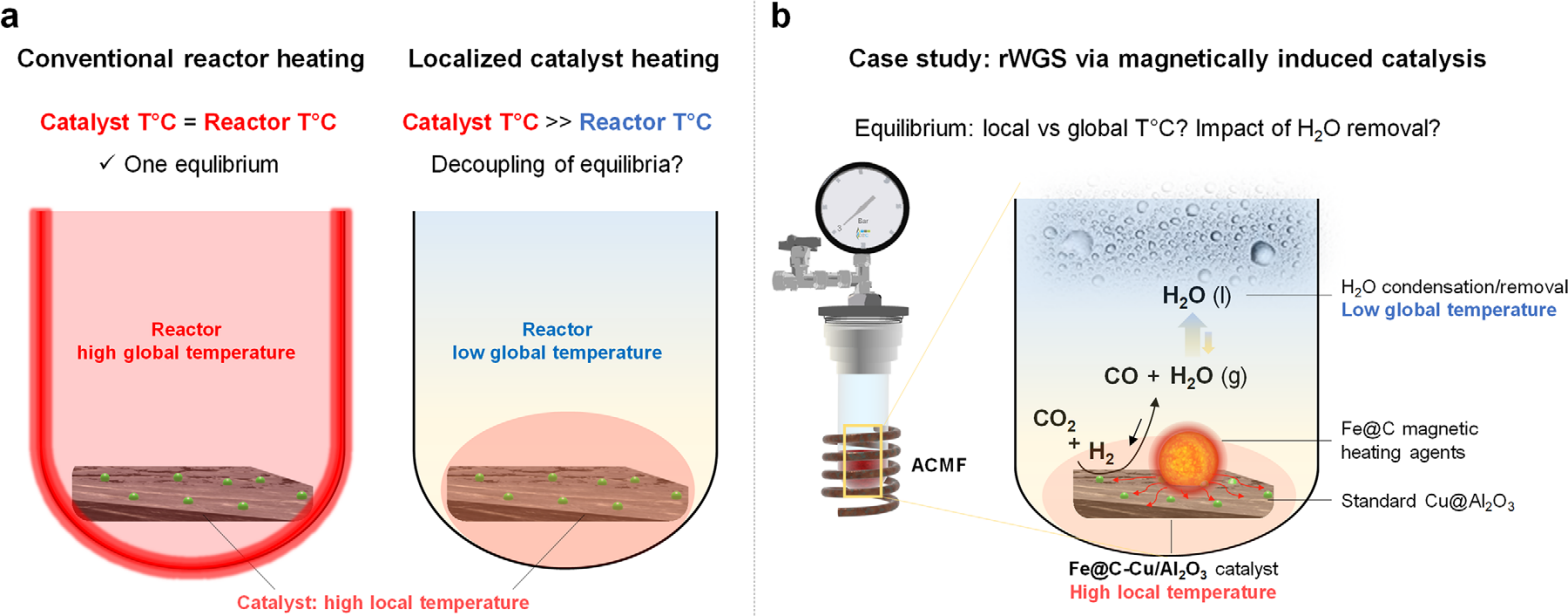
Illustration of the conceptual approach of this study: Can localized catalyst heating improve equilibrium‐limited endothermic reactions? (a) Schematic illustration of reactors with conventional heating and with localized catalyst heating. (b) Our approach to magnetically induced rWGS and associated research questions.

In this context, many opportunities may arise when catalysts are activated in a localized manner, and not anymore by conventional heating relying on the transfer of thermal energy through reactor materials and bulk reaction mixtures [10, 11, 12]. In particular, we hypothesize that the temperature gradients generated by the selective and localized heating of catalyst materials in a colder reactor environment may enable the decoupling of local and bulk equilibria, thereby improving the performance of equilibrium‐limited endothermic reactions (Figure 1a).

To explore this hypothesis, we selected the rWGS as an equilibrium‐limited, endothermic (∆H = +42.1 kJ/mol) reaction that requires high temperatures (500‐800°C) to reach relevant CO yields [7, 8, 9]. It is a crucial transformation for the production of syngas, a key feedstock for various industrial applications, including the manufacture of fuels, chemicals, and fertilizers, and thus a highly relevant case study [1, 13, 14, 15]. In the past decade, significant effort has been dedicated to the development of strategies to improve the efficiency of the rWGS and milden operating conditions [1, 2, 16, 17, 18, 19, 20]. Magnetic induction was selected as a technology enabling the direct and selective heating of catalysts owing to its proven potential to enable localized, rapid, and energy‐efficient heating of appropriately‐designed catalysts [10, 12, 21] for both liquid‐phase [22, 23, 24, 25, 26, 27] and gas‐phase reactions [6, 28, 29, 30, 31, 32]. It also uses electricity to provide thermal energy input into catalytic processes, potentially enabling the incorporation of renewable energy sources into the chemical value chain. Notably, two very recent studies probed the potential of magnetically induced catalysis for the rWGS using specifically designed magnetic nanoparticle catalysts (CoPd/Co@C [33] and FeCo@FeCoO_x_@C [34]). CO yields surpassing theoretical gas phase compositions calculated from reactor temperatures were reported, suggesting that reactions may occur at higher temperatures at the catalyst surface.

In the present study, a key research objective is to reveal whether localized catalyst heating as provided by magnetically induced catalysis (MICat) can enable the decoupling of local and bulk reaction equilibria in equilibrium‐limited endothermic reactions, or more specifically whether the local temperature (catalyst surface) or the global temperature (reactor) is governing the equilibrium product composition of the rWGS [10, 12]. As a consequence of the strong temperature gradients between the locally‐heated catalyst and the colder reactor environment, water condensation may occur in situ and shift the equilibrium toward CO formation via Le Chatelier's principle. Interestingly, in situ water removal proved effective to enhance the hydrogenation of CO_2_ to methanol, [35] or hydrocarbons [36, 37, 38, 39], but its potential impact on the rWGS (magnetically induced or not) has been so far overlooked. We set our goal to explore the occurrence and potential impact of the interlinked effects of equilibria decoupling and water removal on rWGS performance using a *standard heterogeneous catalyst* modified for magnetic induction (Figure 1b).

## 2 Results and Discussion

The major requirement for catalyst design was to grant a standard rWGS catalyst with alternating current magnetic fields (ACMF)‐induced magnetic heating capabilities without altering its intrinsic reactivity. While the functionalization of the rWGS catalyst's surface with magnetic heating agents following an approach recently reported by our team [23, 27] appears attractive, magnetic nanoparticles containing Fe [40], Co [41], and Ni [42] are known for their Fischer–Tropsch and CO_2_ methanation activity. Herein, previously‐reported iron carbide nanoparticles(ICNPs) with excellent magnetic heating capabilities were selected as heating agents [29], and coated with carbon to eliminate their intrinsic CO_x_ hydrogenation activity.

Therefore, 12 nm ICNPs (see Figure S1 for preparation and characterization) were dispersed on ball‐milled sodium chloride (NaCl), and subjected to methane chemical vapor deposition (CVD) at 700°C for 5 min (See Supporting Information for detailed protocol) [43]. After CVD, NaCl was dissolved by washing with water, and carbon‐coated NPs were obtained as a black powder (82.3 wt% Fe content as determined by ICP‐OES, Table S1).

**FIGURE 2:**
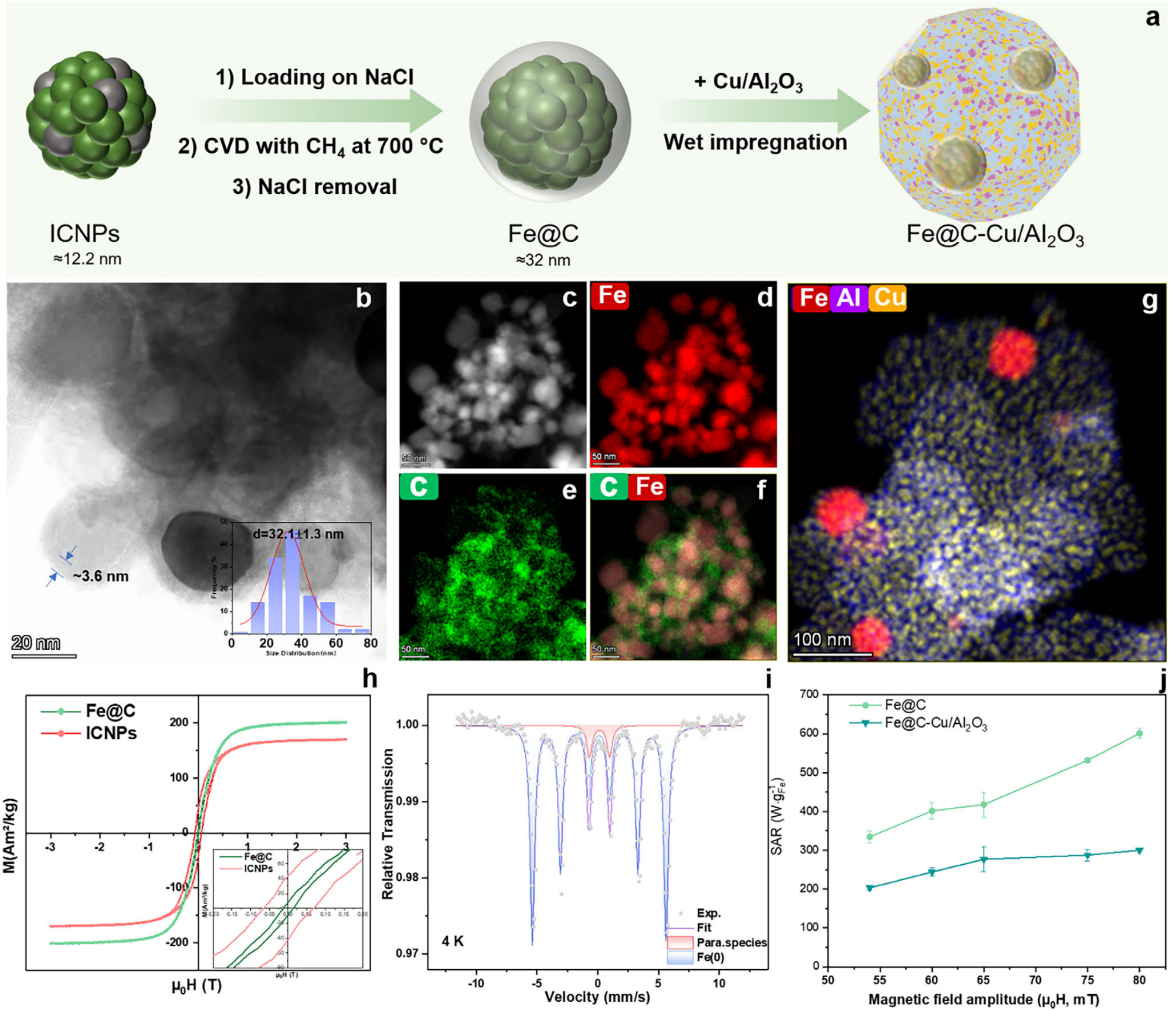
Synthesis and characterization of Fe@C and Fe@C‐Cu/Al_2_O_3_. (a) Schematic illustration of the synthesis process. (b) HRTEM image and size distribution of Fe@C. (c–g) STEM‐HAADF image and corresponding EDX elemental maps showing the spatial distribution of elements in Fe@C (c–f) and Fe@C‐Cu/Al_2_O_3_ g). (h) VSM (300 K) curves of ICNPs and Fe@C. (i) Low‐temperature (4 K) Mössbauer spectrum of Fe@C. (j), Heating power measurements at 350 kHz for Fe@C and Fe@C‐Cu/Al_2_O_3_.

Characterization by electron microscopy revealed a substantial increase in NPs size from 12 nm to 32 nm along with the formation of a carbon shell (∼3.6 nm) (Figures 2b–f and S2–S4). Raman spectroscopy showed the typical D‐G bands, confirming the presence of carbon on the NPs (Figure S5). Interestingly, powder x‐ray diffraction (XRD, Figure S6), Mössbauer spectroscopy (Figure 2i and Table S2), and x‐ray photoelectron spectroscopy (XPS, Figure S7) evidenced the reduction of the iron carbide phases Fe_2.2_C and Fe_5_C_2_ into body‐centered cubic Fe(0) under CVD conditions, as could be expected from the Fe─C phase diagram [44]. Thus, the prepared material consists of carbon‐coated Fe(0) NPs, and will be noted Fe@C NPs. The characterization of magnetic properties by vibrating sample magnetometry (VSM) showed an increase in magnetization at saturation (Ms = 196.3 A m^2^ kg_Fe_^−^^1^) and decrease in coercive field (Hc = 15 mT) in Fe@C NPs as compared to ICNPs (Ms = 170.1 A m^2^ kg_Fe_^−^^1^ and Hc = 68 mT), consistent with the reduction of iron carbide to metallic iron (Figure 2h). Notably, while ICNPs and Fe(0) NPs are air‐sensitive, the carbon shell effectively protected Fe(0) NPs against oxidation for at least 2 weeks, as demonstrated by the stable Ms and absence of exchange bias in VSM analysis (Figure S8) [29], and supported by the absence of Fe_x_O_y_ phases in Raman spectroscopy (Figure S5). The specific absorption rate (SAR, i.e., magnetic heating power) of Fe@C was determined by calorimetric measurements (ACMF, 350 kHz, 54–80 mT, see detailed protocol in the Supporting Information), showing a fairly linear increase in SAR as a function of ACMF amplitude. A maximum SAR of 600 W g^−^^1^ was reached at 80 mT, lower than that of starting ICNPs (3100 W g^−^^1^ at 80 mT, Figures 2j and S9), consistent with the reduced coercivity of the Fe@C NPs. Notably, Fe^0^@C NPs prepared by coating directly Fe(0) NPs of similar size with carbon resulted in materials with substantially poorer magnetic properties (Figure S10).

As a standard rWGS catalyst, Cu/Al_2_O_3_ was synthesized following a previously reported method involving the co‐precipitation of Cu(NO_3_)_2_ and Al(NO_3_)_3_ and reduction of the resulting material (see detailed protocol in Figures S11–S13 and Table S3 for characterization) [45]. ICP‐OES (Table S1) determined Cu and Al contents of 63.8 wt% and 9.5 wt%, respectively. The Cu 2p XPS spectrum (Figure S11) showed that Cu species are predominantly in the metallic state (Cu 2p_3/2_ at 932.5 eV), along with Cu^2+^ species (Cu 2p_3/2_ at 933.7 eV). Cu^0^ and Cu^+^ being difficult to differentiate in Cu 2p XPS, the predominance of Cu^0^ was also confirmed by XRD (Figure S12). These results are consistent with literature reports [45].

The immobilization of Fe@C NPs on Cu/Al_2_O_3_ (17 wt% loading) was achieved by wet impregnation into toluene and THF (see SI for detailed protocol). The resulting Fe@C‐Cu/Al_2_O_3_ material was characterized by nitrogen physisorption experiments (Table S3), giving a Brunauer–Emmet–Teller (BET) specific surface area of 88 m^2^ g^−1^. This value is lower than that of starting Cu/Al_2_O_3_ material (106 m^2^ g^−1^), as expected due to the decoration of the material with Fe@C. High‐angle annular dark field scanning transmission electron microscopy with energy dispersive x‐ray spectroscopy (HAADF‐STEM‐EDX) evidenced a fairly good dispersion of the Fe@C NPs at the surface of Cu/Al_2_O_3_, with a close proximity between Fe@C NPs and Cu NPs (Figure 2g). XRD analysis of the Fe@C‐Cu/Al_2_O_3_ material showed the expected diffraction patterns characteristic of Fe(0) and Cu(0) phases (Figure S6). Elemental analysis by ICP‐OES revealed the distribution Cu = 56.2 wt%, Fe = 11.9 wt% and Al = 12.4 wt%, well in agreement with theoretical expectations (Table S1). Fe@C‐Cu/Al_2_O_3_ possessed a promising SAR (300 W g^−^^1^ at 80 mT), despite an expected decrease in heating power resulting from the immobilization of the Fe@C NPs on Cu/Al_2_O_3_ (Figure 2j).

Reactions were performed in batch mode using thick‐walled borosilicate glassware (43.5 mL Fisher‐Porter bottles) under H_2_/CO (ratio 3/2, total pressure at room temperature 5 bar) for 2 h, using either conventional heating or magnetic induction with a commercial copper coil (*f* = 350 Hz, tunable field amplitude μ_0_H_max_, Figure S14). Reactions with conventional heating at temperatures >200°C were conducted in 45 mL stainless‐steel autoclaves fitted with glass inlets. CO_2_ conversion and gaseous product yields were determined by GC‐TCD and FID calibrated using a certified gas mixture (see Figures S15 and S16 for representative examples of GC chromatograms). The potential presence of liquid products was systematically checked by washing the Fisher‐Porter bottle with acetone and injecting the resulting mixture into GC‐MS. In all cases, only water could be detected as a liquid product (see Figure S17 for a representative example).

**FIGURE 3:**
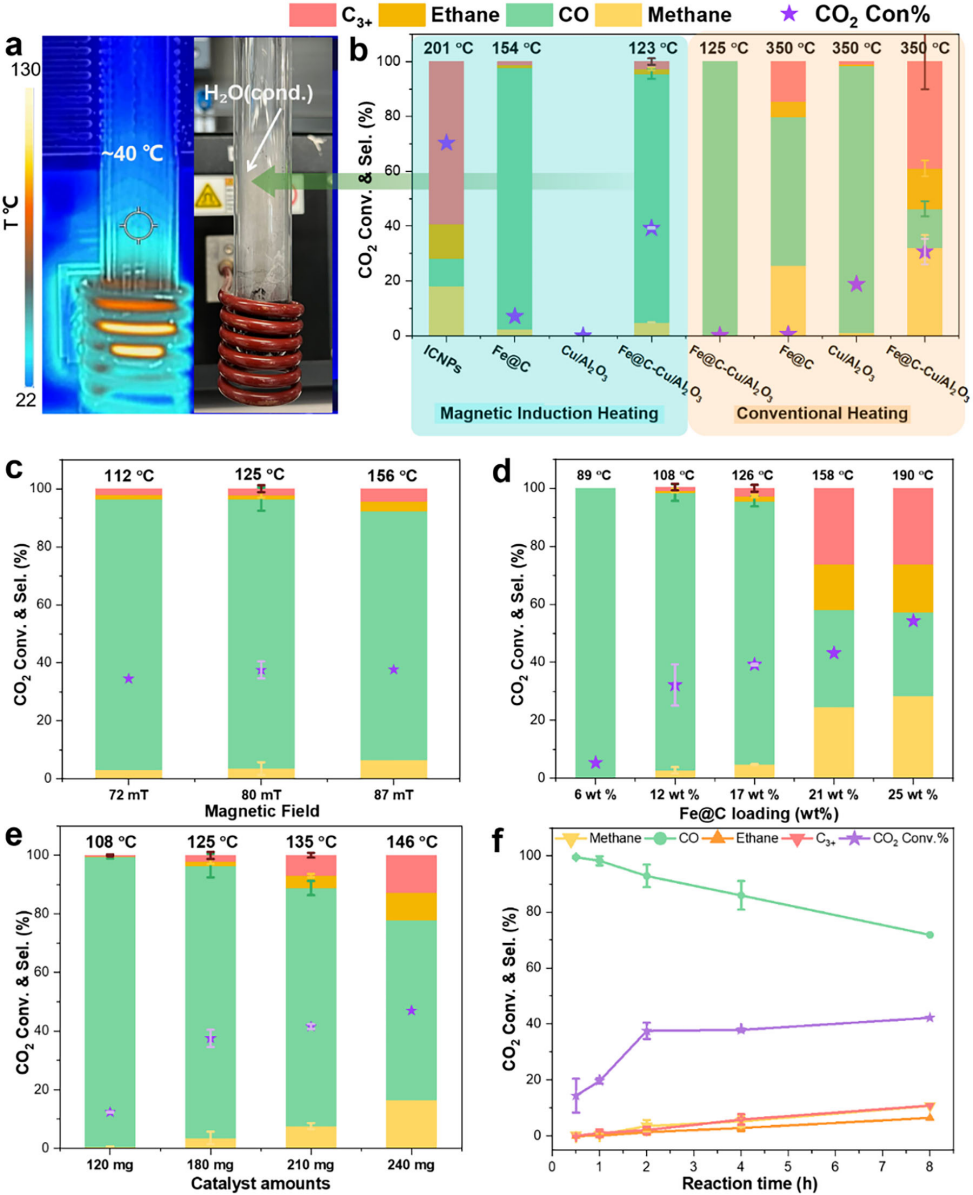
Study of the rWGS using various catalysts and reaction conditions. (a) Infrared (IR) image and corresponding photograph of the reactor under magnetocatalytic conditions; (b) Comparison of the performance of different catalysts under magnetic induction heating (350 kHz, μ_0_H = 80 mT) and conventional heating (125 and 350°C); (c) impact of ACMF amplitude; (d) impact of Fe@C loading on Cu/Al_2_O_3_; (e) impact of catalyst loading in the reactor; (f) time profile of the rWGS under optimized magnetocatalytic conditions using Fe@C–Cu/Al_2_O_3_. Reaction conditions: Catalyst (30 mg Fe@C,30 mg ICNPs, 180 mg Fe@C–Cu/Al_2_O_3_ or 150 mg Cu/Al_2_O_3_), MICat or conventional heating, 5 bar with H_2_/CO_2_ = 3:2, 2 h (for b–e).

Using pristine ICNPs under magnetic induction at a field amplitude of 80 mT, a high CO_2_ conversion (70%) and selectivity toward hydrocarbons (90%) was observed (Figure 3b), consistent with previous reports [39]. The carbon coating in Fe@C NPs effectively suppressed most reactivity (CO_2_ conversion 5%), and enabled a high CO selectivity (95%). Satisfyingly, using the multifunctional Fe@C‐Cu/Al_2_O_3_ catalyst resulted in excellent CO_2_ conversion (39%) and CO selectivity (91%), far superior to the performance of Fe@C NPs alone. These results indicate qualitatively that, although Fe@C has a minor contribution toward the rWGS reaction, its primary role is to provide thermal energy to the more catalytically active Cu/Al_2_O_3_ material.

Under these conditions, the heat dissipated by the Fe@C NPs resulted in a temperature of 123°C determined by an infrared camera at the surface of the Fisher–Porter bottle *in contact with the catalyst bed* (Figures 3a and S18). This estimated reactor temperature is a useful point of comparison with conventional heating approaches involving the heating of the whole reactor environment at a specific temperature. However, it is important to notice that the whole Fisher–Porter bottle is not at 123°C, and that there is a temperature gradient from the hot catalyst bed to the reactor environment (see Figure 3a). The local temperature at the catalyst surface was estimated in the 287–327°C range by placing Fe@C‐Cu/Al_2_O_3_ in solvents of known boiling points and monitoring bubble formation upon ACMF exposure (Table S4). In addition, the temperature of the magnetically‐heated material was estimated by inserting a fiber optic temperature sensor directly in the catalyst bed (Figure S19 and see Supporting Information description for details). The temperature of the catalyst bed rose very rapidly upon application of the ACMF, and reached a plateau at ca. 290°C after 1 min (Figure S20), consistent with the [287–327°C] range derived from the solvent boiling method. While both temperature estimation methods have their limitations and likely underestimate the real catalyst surface temperature under magnetic induction slightly, they demonstrate that the localized heating of a rWGS catalyst by magnetic induction generates strong temperature gradients within the reactor, enabling the reaction to proceed at high temperature at the catalyst surface while the reactor environment is much colder.

Strikingly, using Fe@C‐Cu/Al_2_O_3_ under conventional heating (Figure 3b) at the same global temperature (125°C) gave negligible CO_2_ conversion (0.1%), while adjusting conventional heating toward a comparable catalyst surface temperature (350°C) resulted in increased CO_2_ conversion (31%) but poor CO selectivity (14%). Cu/Al_2_O_3_ did not heat upon exposure to the ACMF and was found inactive under magnetocatalytic conditions.

The energy input required for the magnetocatalytic process using the Fe@C‐Cu/Al_2_O_3_ catalyst under standard conditions was determined and compared to the energy input required for conventional heating at 350°C (see the section “Energy Consumption Analysis” of the Supporting Information). Magnetically activated Fe@C‐Cu/Al_2_O_3_ consumed 0.5 MJ energy to deliver 36% yield of CO in 2 h, while for the same reaction time, conventional heating consumed 1.6 MJ to give much lower catalytic performance (4.6% yield of CO). In addition, 80 min of heating was necessary for the autoclave to reach 350°C, while magnetically induced activation was almost instantaneous. As a result, the energy efficiency toward product formation is ca. 40 times higher with magnetically induced catalysis than with conventional heating at 350°C, even under these nonoptimized laboratory conditions.

Varying the ACMF amplitude from 72 mT to 87 mT impacted the reactor temperature (from 112 to 156°C), and the CO selectivity declined at high amplitude (86% at 87 mT) (Figure 3c). An ACMF amplitude of 80 mT was found to be a good compromise to maximize CO_2_ conversion and CO selectivity. Similarly, increasing the Fe@C NPs loading on Cu/Al_2_O_3_ from 17 wt% to 21–25 wt% led to higher reactor temperatures (158 and 190°C, respectively), with higher CO_2_ conversions (from 39% to 54%) being reached at the expense of CO selectivity (from 91% to 29%). Decreasing the Fe@C NPs loading below 17 wt% resulted in reduced reactor temperatures and catalytic activity, as can be expected from a decrease in the amount of heating agents (Figure 3d). Variation of the loading of Fe@C‐Cu/Al_2_O_3_ in the reactor resulted in a similar trend (Figure 3e). The observed loss of CO selectivity with increasing reactor temperature is presumably due to a decrease in the magnitude of the temperature gradient. As shown in Figure 3f, a time profile recorded under optimized conditions (17 wt% Fe@C NPs loading, 80 mT, 180 mg catalyst) revealed an apparent first‐order reaction with a CO_2_ conversion reaching a plateau (ca. 40%) after 2 h, suggesting that the equilibrium is reached. Notably, the CO selectivity is excellent after 2 h of reaction (93%), but declines slowly upon extension of the reaction time (86% at 4 h, 72% at 8 h) due to the formation of hydrocarbons via a Fischer–Tropsch‐like mechanism.

Most intriguingly, the CO yield (36%) observed under optimized magnetocatalytic conditions is substantially higher than the expected gas phase thermodynamic equilibria, not only at 125°C (3%, reactor temperature at *contact point* with the catalyst), but also at 300°C (16%, estimated catalyst temperature). This can be attributed to the formation of a film of liquid water on the cold walls of the Fisher–Porter bottle under reaction conditions (Figure 3a). Performing the same reaction under ACMF while heating the Fisher–Porter walls with a heat gun to limit water condensation resulted in a CO yield of 23% (Figure S21 and Table S5), much closer to the theoretical gas phase equilibrium value at 300°C (Figure 5a).

This result indicates that in magnetically induced rWGS, the gas phase thermodynamic equilibrium is governed by the catalyst/local temperature and not by the reactor/global temperature, demonstrating effective decoupling of local and bulk reaction equilibria.

**FIGURE 4:**
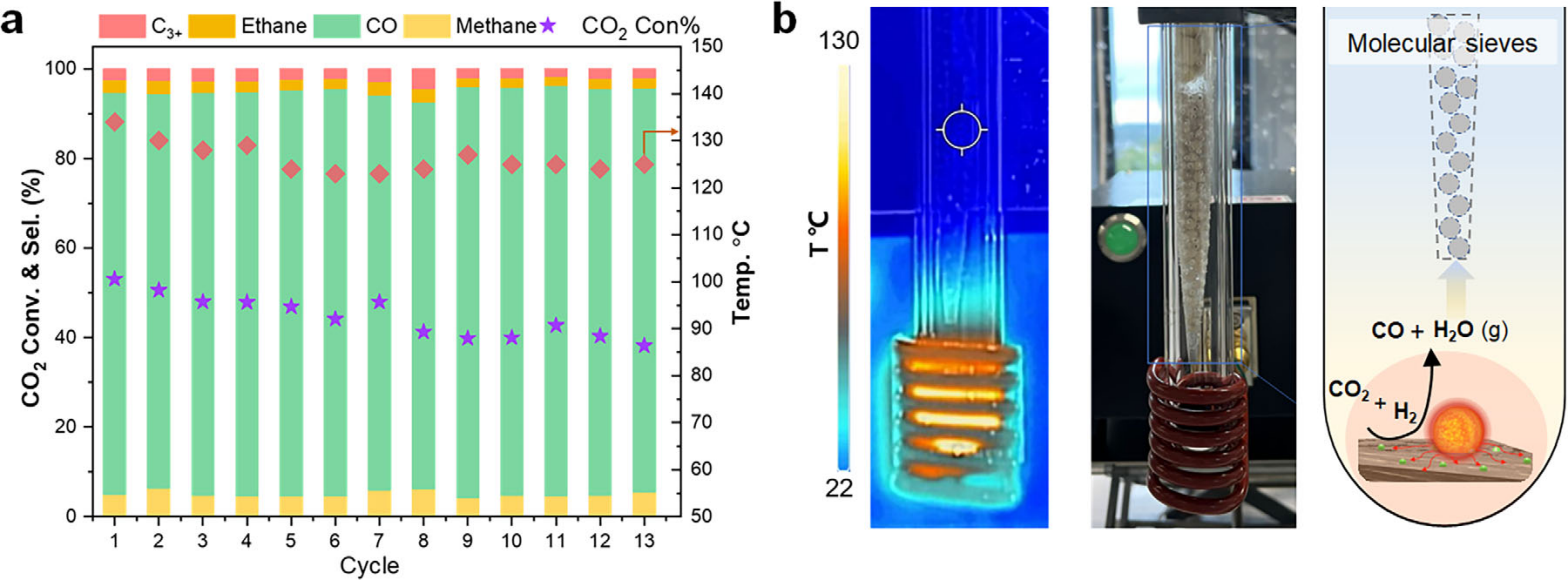
Recyclability of Fe@C‐Cu/Al_2_O_3_ in magnetically induced rWGS. **a**) Recyclability tests of Fe@C‐Cu/Al_2_O_3_ in magnetically induced rWGS in the presence of 3 Å molecular sieves. Reaction conditions: Fe@C‐Cu/Al_2_O_3_ (180 mg, 17 wt%), total pressure 5 bar (H_2_:CO_2_ = 3:2), 350 kHz, μ_0_H = 80 mT, 2 h, 43.5 mL Fisher‐Porter bottle. b) Photograph of the Fisher‐Porter bottle reactor, including the 3 Å molecular sieves cartridge.

**FIGURE 5:**
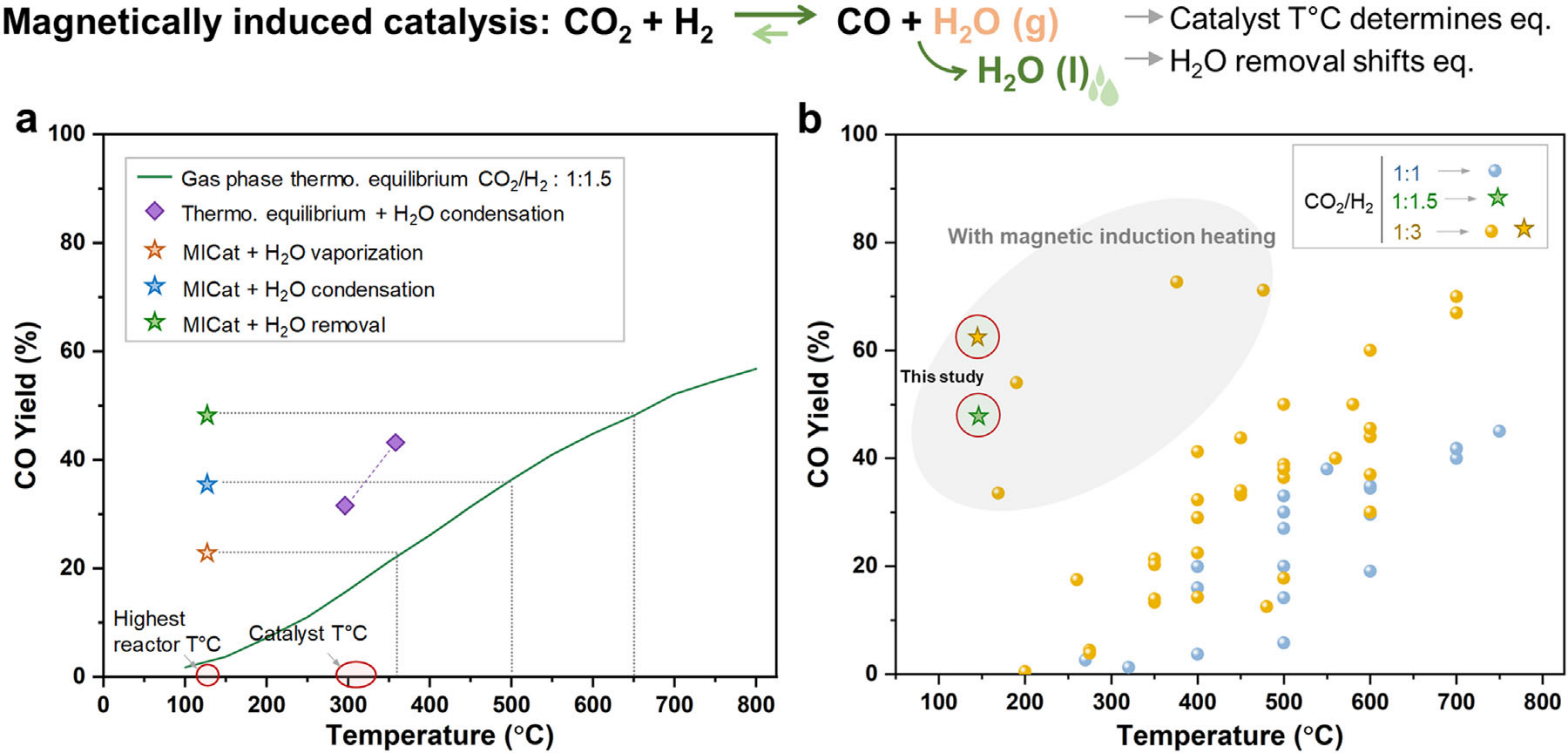
CO yield as a function of temperature and comparison with literature data. (a) Plot of CO yield as a function of temperature. The stars represent the data obtained in this study. The green line shows the gas‐phase thermodynamic equilibrium, while the violet points indicate the thermodynamic equilibrium while accounting for water condensation (see Supporting Information for details). (b) Comparison of the best result of this study (circled stars, MICat + in situ water removal) with literature data (individual‐colored disks, see Table S6 for details).

In addition, under standard conditions (i.e., without additional heating of the reactor wall), MICat allows the condensation of water on the relatively cold walls of the Fisher‐Porter bottle (Figure 3a), thereby shifting the reaction thermodynamic equilibrium via the Le Chatelier principle and enabling even higher CO yields. Calculation Estimation of the thermodynamic equilibrium composition while accounting for the condensation of water vapor into liquid water gave CO yields of 31% at 300°C and 43% at 350°C (see Figure 5a and Supporting Information for details), consistent with the 36% CO yield reached in MICat. While such simplified calculations do not capture the full complexity of the system (solid‐liquid‐gas interface, non‐isothermal conditions), they at least qualitatively illustrate the impact of water condensation on the shift of the reaction equilibrium. The cold reactive environment enabled even the active in situ removal of water via adsorption, exemplified here through the addition of a cartridge containing 3 Å molecular sieves above the catalyst bed (cartridge at ∼ r.t., see Figures 4 and S22 for details). The very hot catalyst surface promotes the transport of water vapor toward the colder reactor environment, where it is adsorbed by the molecular sieves. This simple approach proved even more effective than simple condensation to shift the thermodynamic equilibrium (48% CO yield with water removal, 36% with water condensation). Under these conditions, a total of 1.68 mmol of water was generated (ca. 30 mg). This is more than 10 times lower than the maximum adsorption capacity of the molecular sieves cartridge (∼400 mg for 2 g of sieves), suggesting that mass‐transfer limitations should be negligible.

The decoupling of local and bulk equilibria, together with the effective removal of water are uniquely linked to the generation of strong temperature gradients between the hot catalyst and the colder reactor environment. The present performance was achieved in batch‐type reactors, and further studies towards specific reactor design enabling appropriate heat and water‐removal management under continuous‐flow operation seem promising.

The recyclability and stability of the magnetically‐heated Fe@C‐Cu/Al_2_O_3_ catalyst were investigated under standard conditions, revealing a rapid decline in CO_2_ conversion after the first cycle (from 38% to 7%, Figure S23). Characterization of the spent Fe@C‐Cu/Al_2_O_3_ material by XRD revealed Cu oxide–related reflections, indicating partial oxidation of metallic Cu (Figure S24). This observation was supported by the analysis of the XPS Cu 2p spectra (Figure S25), which evidenced an increased Cu^2^^+^ fraction as compared to the fresh catalyst as well as to the spent catalyst recovered from experiments performed in the presence of molecular sieves. In addition, BF‐STEM and HAADF‐STEM‐EDX (Figure S26) showed substantial growth and aggregation of Cu nanoparticles after one reaction cycle without active water removal, whereas the particle size remains largely preserved even after 13 cycles when molecular sieves were used (Figure S29). These results point to a catalyst deactivation due to aggregation and water‐induced oxidation. Deactivation of Cu‐based rWGS catalysts through oxidation by water is a well‐known issue, especially in batch mode [1, 35].

While treatments under H_2_ proved unsuccessful to fully regenerate the catalyst reactivity (Figure S23), the in situ removal of the water produced in the rWGS reaction by molecular sieves was found to be very promising. Under these conditions, the CO_2_ conversion could be maintained in the 40%–53% range for 13 cycles of 2 h, with only a minor decrease with time (Figure 4). A similar behavior was observed for 1 h cycles (Figure S27), that is, at conversion levels below the equilibrium. The reactor temperature was found to be perfectly stable throughout the cycles, illustrating the stability and robustness of the Fe@C NPs heating agents. Post‐recycling characterization was carried out using XRD, VSM, XPS, Raman, and HRTEM. The Fe@C‐Cu/Al_2_O_3_ catalyst exhibited a small increase in *M_s_* and no observable exchange bias in the VSM analysis after the reaction, indicating fairly stable magnetic properties (Figure S28). The morphology of the sample remained largely unchanged (Figure S29). EDX mapping after 13 catalytic cycles revealed slight agglomeration and growth of the Cu nanoparticles, which may be responsible for the minor decrease in catalytic performance throughout cycles. As shown by the XPS results (Figure S25), the presence of the molecular sieve effectively preserved the Cu^0^ phase during cycling and prevented oxidation by water, with only a minor decrease in the Cu^0^ content from 83% to 80%. Raman spectroscopy did not reveal noticeable change in the characteristic D and G bands of the carbon shell (Figure S30), while XRD (Figure S24) and high‐resolution Fe 2p XPS (Figure S31) showed the preservation of the metallic Fe core and the absence of iron oxide or carbide phases. These results are consistent with the excellent selectivity toward CO, and demonstrate that the carbon shell is robust and conserves its integrity under the reaction conditions applied.

## 3 Conclusion

To conclude, we developed a synthetic methodology for air‐stable and ferromagnetic carbon‐coated iron NPs that are used as nonreactive heating agents to activate a standard heterogeneous rWGS catalyst by magnetic induction. Under optimized magnetocatalytic conditions, the Fe@C‐Cu/Al_2_O_3_ multifunctional catalytic system proved robust and delivered maximum CO yields of 48% (91% selectivity; H_2_:CO_2_ = 1.5:1) and 62% (90% selectivity; H_2_:CO_2_ = 3:1) at a reactor temperature of 123°C (highest temperature at contact point with the catalyst bed) and a catalyst temperature estimated to 300°C (Figure 5a). Strikingly, reaching the same CO yields in a conventionally heated gas phase rWGS would require a reactor temperature of ca. 650°C. These are among the best rWGS performances at low temperature reported to date (Figure 5b).

Our results show that the striking enhancement as compared to the gas phase equilibria predicted by Gibbs free energy minimization at the same temperatures (CO yield of 3% at 125°C, 16% at 300°C) originates from two main interlinked factors: (1) the gas phase equilibrium composition is determined by the estimated local catalyst temperature and not by the reactor temperature, meaning that local and bulk reaction equilibria are successfully decoupled, and (2) the in situ removal of water from the reactive environment is promoted by the generated temperature gradients, which helps shifting the gas phase thermodynamic equilibrium and stabilizing the Cu‐based active species. While the occurrence of nonthermal effects from the activation by ACMF [46] cannot be ruled out at this stage, our data and observations can be rationalized consistently on the basis of thermodynamic considerations of the rWGS equilibria.

These findings substantiate the potential of localized catalyst heating to enable the rWGS reaction under particularly mild conditions, and may potentially bear significant implications for the operation of other equilibrium‐limited endothermic reactions.

## Conflicts of Interest

The authors declare no conflicts of interest.

## Supporting information




**Supporting File 1**: anie71297‐sup‐0001‐SuppMat.pdf.

## Data Availability

Data are available in the article's Supporting Information. Source data are available for download on Edmond (open data repository of the Max Planck Society) at https://doi.org/10.17617/3.XVFJMF.
