# Supplementary material for: Low‐Temperature Reverse Water–Gas Shift Enabled by Magnetically Induced Catalysis
Source: Angew Chem Int Ed Engl. 2026 Jan 28;65(16):e23576. doi: 10.1002/anie.202523576 (PMC13080426; doi:10.1002/anie.202523576)
Supplement: Supplementary file 1 — Supporting File 1: anie71297‐sup‐0001‐SuppMat.pdf. [file ANIE-65-e23576-s001.pdf]

Supplementary Information

Low Temperature Reverse Water-Gas Shift Enabled by Magnetically Induced Catalysis

Junhui Hu, Lise Marie Lacroix, Jacob Johny, Sourav Ghosh, Elisabeth Hannah Wolf, Jeongmin Ji, Sheng-Hsiang Lin, Manisha Durai, Alin Benice Schöne, Walid Hetaba, Holger Ruland, Walter Leitner*, Alexis Bordet*

J. Hu, Dr. J. Johny, Dr. E. H. Wolf, Dr. J. Ji, Dr. S.-H. Lin, M. Durai, A. B. Schöne, Dr. W. Hetaba, Dr. H. Ruland, Prof. Dr. W. Leitner, Dr. A. Bordet
Max Planck Institute for Chemical Energy Conversion
Stiftstraße 34-36, 45470 Mülheim an der Ruhr (Germany)
[*] E-mail: walter.leitner@cec.mpg.de; alexis.bordet@cec.mpg.de

J. Hu, M. Durai, Prof. Dr. W. Leitner
Institut für Technische und Makromolekulare Chemie, RWTH Aachen University
Worringerweg 2, 52074 Aachen (Germany)

Dr. L. M. Lacroix, Dr. S. Ghosh
Laboratoire de Physique et Chimie des Nano-Objets Université de Toulouse, LPCNO, INSA, UPS CNRS-UMR 5215
135 Avenue de Rangueil, 31077 Toulouse (France)

Dr. L. M. Lacroix
Institut Universitaire de France (IUF)
103 boulevard Saint Michel, 75005 Paris (France)

Dr. J. Ji
Current address: Department of Materials Science and Engineering, Gwangju Institute of Science and Technology (GIST)
61005 Gwangju (Republic of Korea)

Materials and Methods

Chemicals and instruments

All syntheses were carried out under an argon atmosphere, utilizing either Schlenk techniques or a glovebox. Solvents were purified using a solvent purification system (MBraun-SPS-7) or dried over activated 3Å molecular sieves, then degassed and stored under argon prior to use. Hexadecylamine (HDA, 99%), Palmitic acid (PA, 99%), Aluminum nitrate nonahydrate (98%), Sodium chloride (99%), Copper(II) nitrate trihydrate (99.9%), and other chemicals were sourced from Sigma-Aldrich. The Bis(amido)iron(II) dimer ($\{Fe[N(SiMe_3)_2]_2\}_2$) was synthesized following established procedures.[26,47] The catalyst synthesis was performed using a ball mill (Retsch MM500), a muffle furnace (Nabertherm P330), and a tubular furnace (Carbolite Gero). Magnetic induction heating experiments were conducted with a UPT-n5 device from Ultraflex Power Technologies, operating at a fixed frequency of 350 kHz and a tunable magnetic field amplitude, along with a 6-turn coil (ID 25 mm x length 42 mm). The magnetic field amplitude remained largely uniform, with variations not exceeding 10%.

Synthesis of carbon-coated iron nanoparticles (Fe@C)

First, ICNPs, (~13 nm) were synthesized via the carbidization of preformed Fe(0) nanoparticles under a CO/H_2 atmosphere, following a previously reported procedure by our group. [23]

In a typical synthesis, Fe (0) NPs were prepared as follows: Inside a glovebox, PA (2.6 mmol, 1332.8 mg) and HDA (2 mmol, 966 mg) were added to a green solution of $\{Fe[N(SiMe_3)_2]_2\}_2$ (0.5 mmol, 1506 mg) in distilled and degassed mesitylene (80 mL) in a Fisher–Porter bottle. The bottle was pressurized with H_2 (4 bar) and heated in an oil bath at 150 °C for 48 h under vigorous magnetic stirring. After cooling to room temperature, the reaction was stopped, and the NPs were recovered by magnetic decantation and washed three times with toluene (3 × 10 mL) and three times with THF (3 × 10 mL), followed by drying under vacuum. Subsequently, Fe (0) NPs (300 mg) were dispersed in mesitylene (25 mL) in a Fisher–Porter bottle, which was then pressurized with a CO/H_2 mixture (3 bar / 2 bar) and heated at 150 °C for 5 days. The resulting NPs were collected by magnetic decantation, washed three times with toluene (3 × 5 mL), and dried under vacuum.

Subsequently, ICNPs (100 mg) were mixed with sodium chloride (50 g, pre-treated by ball milling at 25 Hz for 2 h), and 50 mL of toluene. The mixture was ultrasonically dispersed for 1 h, vacuum-dried overnight to remove toluene, and then transferred into a silica boat. The boat was placed in an electric tube furnace and heated to target temperatures of 300, 500, 600, and 700 °C at a heating rate of 15 °C min^{-1} under a flow of Ar (200 mL min^{-1}). Subsequently, the sample underwent heat treatment in a mixed gas atmosphere of methane (99.9% purity, 100 mL min^{-1}), Ar (200 mL min^{-1}), and H_2 (100 mL min^{-1}) at the same growth temperatures (300, 500, 600, and 700 °C) for 5 minutes. The samples were then labeled as Fe@C-300, Fe@C-500, Fe@C-600, and Fe@C-700, respectively.

After cooling to room temperature, the resulting nanoparticles were washed with degassed water, vacuum-dried overnight, and collected using a magnet. The final products were stored in a glovebox to prevent oxidation. Fe^0@C NPs were synthesized using the same approach, performing the carbon coating directly on the preformed Fe(0) NPs.

Synthesis of Cu/Al_2O_3

To prepare the coprecipitated Cu/Al_2O_3 catalysts, a literature procedure was followed. [45] 15 g (0.062 mol) of $Cu(NO_3)_2{\cdot}3H_2O$ and 11.65 g (0.031 mol) of $Al(NO_3)_3{\cdot}9H_2O$ were dissolved in 500 mL of deionized water. A mixed solution of NaOH (1.0 M) and Na_2CO_3 (1.0 M) was prepared as the precipitating agent and added dropwise to the metal precursor solution, while maintaining the pH at ~9.5. The resulting suspension was stirred overnight at 75 °C. The resulting precipitate was collected by filtration and washed with deionized water until the filtrate reached neutrality (pH ~7). The sample was dried at 110 °C for 12 h under vacuum, ground into fine powders, and calcined at 600 °C (5 °C·min^{-1}) in a muffle furnace for 6 h, and labeled as CuO/Al_2O_3. Next, 3 g of the sample was placed in a silica boat and heated in an electric tube furnace to 300 °C at a rate of 1 °C·min^{-1} under a continuous H_2 flow (60 mL·min^{-1}) for 5 h, and labeled as CuAl-H_2-1st. After cooling to room temperature, the material was exposed to an air flow (200 mL·min^{-1}) for 0.5 h and labeled as CuAl-air-exposed. **Caution:** During air exposure, a significant amount of heat was released, increasing the surface temperature to approximately 300 °C (measured by IR camera). Subsequently, 1.3 g of the air-exposed sample underwent a second annealing under identical conditions (300 °C, 1 °C·min^{-1} heating rate, 60 mL·min^{-1} H_2 flow), maintaining 300 °C for 5 h. To prevent oxidation, the final product was stored in a glovebox and labeled as Cu/Al_2O_3

Synthesis of Fe@C-Cu/Al_2O_3

30 mg of Fe@C were mixed with 150 mg of Cu/Al_2O_3 in 6 mL of a solvent mixture (THF:toluene = 1:2, v/v). The suspension was ultrasonicated for 1 h to achieve homogeneous dispersion, followed by overnight vacuum drying to remove the solvent. The resulting solid was ground into a uniform black magnetic powder and denoted as Fe@C-Cu/Al_2O_3.

Characterization

The size and morphology of the as-synthesized nanoparticles were analyzed using Transmission Electron Microscopy (TEM). TEM grids were prepared by drop-casting a colloidal solution of the nanoparticles onto gold grids coated with amorphous carbon. Conventional bright-field images, high-resolution TEM (HR-TEM), scanning TEM with high-angle annular dark-field imaging (STEM-HAADF), and energy-dispersive X-ray spectroscopy (EDX) were acquired using a Thermo Scientific Talos F200X microscope equipped with a field emission gun (FEG) operating at 200 kV.

X-ray diffraction (XRD) measurements were carried out on a PANalytical Empyrean diffractometer using Co-Kα radiation (λ = 0.1789 nm) at 45 kV and 40 mA.

Magnetic measurements were conducted with a Vibrating Sample Magnetometer (VSM, Quantum Device PPMS Evercool II). Magnetization curves were recorded up to +/- 3T at 300K and 5K after the sample was cooled down under an external magnetic field µ0Hext of +3T. Both XRD and VSM studies were performed on compact powder samples and diluted assemblies, which were prepared and sealed under an argon atmosphere.

Mössbauer spectra were collected using a spectrometer with a conventional constant-acceleration γ source (57Co in a Rh matrix, 1.8 GBq). Sample temperature was maintained using a Cryogen-Free Magnet (CFM) with an integrated variable temperature insert (VTI) for zero-field measurements. The minimum experimental linewidth was 0.24 $mm\cdot s^{-1}$, and isomer shifts are reported relative to α-iron at 300 K. Data analysis was performed using the MX program written by Dr. Eckhard Bill.

X-ray photoelectron spectroscopy (XPS) experiments were conducted with monochromatized Al-Kα radiation (1486.6 eV) and a Phoibos NAP-150 hemispherical analyzer from SPECS GmbH. High-resolution spectra were collected at a pass energy of 20 eV. The instrument work function was calibrated to yield a binding energy of 83.96 eV for the Au $4f_{7/2}$ peak of metallic gold. The base pressure of the instrument was maintained at 5×10^{-8} mbar.

The Brunauer−Emmett−Teller (BET) surface area, Barrett−Joyner−Halenda (BJH) mesopore volume, and DFT micropore volume were determined from N_2 physisorption measurements conducted on a Micromeritics 3Flex apparatus at liquid nitrogen temperature, over a relative N_2 pressure range of 10^{-5} to 0.99. Approximately 100 mg of each sample was pre-dried at 120 °C (with a temperature ramp of 2 °C min^{-1}) under vacuum (<10^{-3} mbar) for 4 h. A leak test was performed before the analysis to ensure system integrity.

The structure of the carbon species on the Fe@C was characterized using a Renishaw in Via Confocal Raman microscope (specific model: Thermo Scientific DXR Raman Microscope (laser 532 nm + 780 nm) range: 400-4000 cm^{-1}), and the spent samples were excited by a 532 nm laser (maximum power: 10 mW, used power: 0.1 mW) over the range of 270–4000 cm^{-1}. The spectral acquisition consisted of ten scans with 64 exposures with each a time of 1s at 0.1 mW laser power. Data acquisition and processing were performed using OMNIC software. For the comparison of different samples, all the peak intensities were normalized to the intensity of the D band.

Hyperthermia measurements

In a typical hyperthermia experiment, ~10 mg of ICNPs, Fe@C and 40 mg of 17 wt% Fe@C–Cu/Al_2O_3 dispersed in 0.3 mL mesitylene was sealed under inert atmosphere in an air-tight tube. The tube was then placed in a calorimeter containing 2 mL deionized water. Temperature was monitored using two probes (top and bottom), and the system was exposed to an alternating magnetic field for 10–40 s, keeping the temperature rise below 20 °C. After exposure, the calorimeter was shaken to ensure uniform temperature, and the temperature rise was determined from the average slope of the $\Delta T/\Delta t$ curve. Specific absorption rate (SAR) values were calculated using the expression:

$$\mathrm{SAR} = \frac{\sum_i C_{pi} m_i}{m_{Fe}} * \frac{\Delta T}{\Delta t}$$

where C_{pi} and m_i are the specific heat capacity and mass of each component: Fe NPs (449 $J\cdot kg^{-1}\cdot K^{-1}$), mesitylene (1750 $J\cdot kg^{-1}\cdot K^{-1}$), water (4186 $J\cdot kg^{-1}\cdot K^{-1}$), and glass (720 $J\cdot kg^{-1}\cdot K^{-1}$). m_{Fe} is the mass of Fe in the materials.

Temperature measurements using a fiber optic temperature sensor

In a typical thermometer experiment, an Omnifit® chromatography column was used as the reactor. Fe@C–Cu/Al_2O_3 (17 wt%, 180 mg) was sealed in the reactor under an inert atmosphere. A fiber optic temperature sensor (OTG-M420) was embedded inside the Fe@C–Cu/Al_2O_3 and connected to a fluoroptic thermometer (m920, LumaSense Technologies). The real-time temperature was monitored using the corresponding temperature acquisition software. To minimize measurement errors, each experiment was repeated at least three times, with a heating duration of 10 min for each measurement.

Catalyst Testing

Magnetically induced catalysis

The coil, manufactured by Ultraflex, was composed of a 6-turn copper solenoid with a height of 42 mm and connected to a 5 kW AC magnetic induction system (Ultraflex) operating at 350 kHz. The RMS field amplitude was adjustable between 0 and 87 mT. The bulk temperature during the reaction was monitored using an infrared (IR) camera.

For product analysis, the Fisher–Porter bottle was connected to a gas chromatography (GC) line. After purging the system to eliminate any residual gases in the tubing, the bottle was opened, allowing the gas phase to be directly injected into the GC. The resulting chromatogram provided the composition and distribution of gaseous products.

Quantitative analysis was based on the integrated peak areas in the chromatograms. Given that different gases exhibit varying response factors with the thermal conductivity detector (TCD), calibration was performed using a certified standard gas mixture containing known concentrations of H_2, CO_2, CO, CH_4, C_2H_6, and Ar (Messer Industriegase GmbH; molar composition: 10% H_2, 20% CO_2, 20% CO, 10% CH_4, 5% C_2H_6, 35% Ar).

In our setup, helium was used as the carrier and reference gas for the TCD. Consequently, compounds with thermal conductivities most different from that of helium exhibit the highest detector response. To quantify this effect, we used the peak area of CO_2 as the internal reference to determine the relative response factors for H_2, CO, CH_4, C_2H_6, and Ar.

The response factors were derived from the chromatogram obtained during the analysis of the certified standard gas mixture. (Figure S15) As expected from their respective thermal conductivities, the detector responses varied significantly. The response correction factors (FC) were calculated as follows:

$$\mathrm{FC(H_2)} = \frac{\%_{H_2} * A(CO_2)}{\%_{CO_2} * A(H_2)}$$

$$\mathrm{FC(CO)} = \frac{\%_{CO} * A(CO_2)}{\%_{CO_2} * A(CO)}$$

$$\mathrm{FC(Ar)} = \frac{\%_{Ar} * A(CO_2)}{\%_{CO_2} * A(Ar)}$$

$$\mathrm{FC(CH_4)} = \frac{\%_{CH_4} * A(CO_2)}{\%_{CO_2} * A(CH_4)}$$

$$\mathrm{FC(C_2H_6)} = \frac{\%_{C_2H_6} * A(CO_2)}{\%_{CO_2} * A(C_2H_6)}$$

Component	Molar %	Average Area	Average FC	FC Uncertainty
H_2	10	1997.8	113.168	±4.403
Ar	35	702279.3	1.125	±0.00560
CO	20	377940.1	1.194	±0.00653
CH_4	10	147523.9	1.530	±0.0209
CO_2	20	451263.3	1	0
C_2H_6	5	107675.4	1.050	±0.0375

Calibration data for the gas chromatograph and calculated correction factors

It is important to note that the standard gas mixture was injected and analyzed repeatedly (15 independent measurements) to ensure the reliability of the response correction factors. The calculated correction factors showed excellent reproducibility, with minimal variation across all measurements, as reflected in the low associated uncertainties.

The initial CO_2 conversion ($X_{CO_2}^{(0)}$), as well as the selectivity to CH_4 ($S_{CH_4}^{TCD}$) and CO (S_{CO}^{TCD}), were determined based on the integration of peak areas obtained from TCD.

$$X_{CO_2}^{(0)} = \frac{\sum_{TCD}(FC_i * A_i)}{\sum_{TCD}(FC_i * A_i) + A_{CO_2}^{TCD}} \quad (i = CO, CH_4)$$

$$S_{CH_4}^{TCD} = \frac{FC_{CH_4} * A_{CH_4}^{TCD}}{\sum_{TCD}(FC_i * A_i)}$$

$$S_{CO}^{TCD} = \frac{FC_{CO} * A_{CO}^{TCD}}{\sum_{TCD}(FC_i * A_i)}$$

Since methane is detectable by both the TCD and flame ionization detector (FID), it was employed as a bridging compound to calibrate the FID response. The FID peak areas were then used to estimate the selectivity of other hydrocarbon products.

$$R_i = \frac{A_i^{FID}}{A_{CH_4}^{FID}} (i = C_2H_6, C_3H_8, \ldots)$$

To quantify the selectivity of hydrocarbons not directly detectable by the TCD, the ratio of their FID peak areas to that of CH_4 was calculated. By using the CH_4 selectivity derived from the TCD as an anchor, the unnormalized selectivity of other hydrocarbons were estimated accordingly.

$$S_i^{raw} = R_i * S_{CH_4}^{TCD}$$

Subsequently, the total unnormalized selectivity was calculated as the sum of all individual unnormalized product selectivity. Each product's selectivity was then normalized relative to this total, yielding the final normalized selectivity values.

$$S_{sum} = S_{CH_4}^{TCD} + S_{CO}^{TCD} + \sum S_i^{raw}$$

$$S_i^{norm} = \frac{S_i^{raw}}{S_{sum}}$$

$$S_{CO}^{norm} = \frac{S_{CO}^{TCD}}{S_{sum}}$$

$$S_{CH_4}^{norm} = \frac{S_{CH_4}^{TCD}}{S_{sum}}$$

Finally, the corrected CO_2 conversion was obtained by adjusting the initial conversion ($X_{CO_2}^{(0)}$) with the ratio of the total selectivity after normalization to that before normalization, thereby accounting for all detectable carbon-containing products.

$$X_{CO_2}^{final} = \frac{X_{CO_2}^{(0)}}{100\ \%} * S_{sum}$$

In a typical experiment, 180 mg of nanoparticles were loaded into a 43.5 mL Fischer–Porter bottle, which was pressurized with a H_2/CO_2 gas mixture (total pressure: 5 bar; molar ratio: 3:2), with or without the addition of 3Å molecular sieves. The bottle was placed at the center of a coil and exposed to an alternating magnetic field with an amplitude of 80 mT for 2 h. The reaction products were subsequently analyzed by gas chromatography. (Figure S16)

Equilibrium Calculation of the RWGS Reaction with Liquid Water

N.B. These calculations are simplified and do not capture the full complexity of our catalytic system ((solid-liquid-gas interface, non-isothermal). Their purpose is to estimate and illustrate conceptually the impact of water condensation on the equilibrium composition observed along the effect of localized heating.

CO_2 (g) + H_2(g) $\rightleftharpoons$ CO(g) + H_2O(g)

CO_2 (g) + H_2(g) $\rightleftharpoons$ CO(g) + H_2O(l)

The reaction thermodynamics are first evaluated for water in the gas phase, then corrected for condensation to liquid water.

1. Thermodynamic Data for Gas-Phase Water:

Species	ΔH (kJ/mol)	ΔG (kJ/mol)	ΔS ($J \cdot mol^{-1} \cdot K^{-1}$)
CO_2(g)	−393.5	−394.4	213.7
H_2(g)	0	0	130.7
CO(g)	−110.5	−137.2	197.7
H_2O(g)	−241.8	−228.6	188.8
H_2O(l)	−285.8	−237.1	69.9

Standard Thermodynamic Data at 298 K

$\Delta H^{\circ}_{RWGS,H_2O(g)}$=$\Delta H^{\circ}_{H_2O(g)}$+ ΔH°_{CO}-$\Delta H^{\circ}_{H_2}$- $\Delta H^{\circ}_{CO_2}$= +41.2 kJ/mol

$\Delta G^{\circ}_{RWGS,H_2O(g)}$=$\Delta G^{\circ}_{H_2O(g)}$+ ΔG°_{CO}-$\Delta G^{\circ}_{H_2}$- $\Delta G^{\circ}_{CO_2}$= +28.6 kJ/mol

$\Delta S^{\circ}_{RWGS,H_2O(g)}$=$\Delta S^{\circ}_{H_2O(g)}$+ ΔS°_{CO}-$\Delta S^{\circ}_{H_2}$- $\Delta S^{\circ}_{CO_2}$= +42.1 $J \cdot mol^{-1} \cdot K^{-1}$

$\Delta G = \Delta H - T * \Delta S$

Assuming the temperature dependence is small, we approximate:

$\Delta H^{\circ}_{298\,K} \approx \Delta H^{\circ}_{T}$

$\Delta S^{\circ}_{298\,K} \approx \Delta S^{\circ}_{T}$

Thus, the reaction Gibbs free energy at temperature T can be estimated by:

$\Delta G^{\circ}_{RWGS,H_2O(g)}(T) \approx \Delta H^{\circ}_{RWGS,H_2O(g)} - T * \Delta S^{\circ}_{RWGS,H_2O(g)}$

$\Delta G^{\circ}_{RWGS,H_2O(g)}(623K)$= +15.0 kJ/mol

$\Delta G^{\circ}_{RWGS,H_2O(g)}(573K)$= +17.1 kJ/mol

2. Condensation Correction for Liquid Water:

Water condenses to liquid in the low-temperature part of the system. The condensation free energy is estimated as:

$\Delta G^{\circ}_{H_2O,cond} = \Delta G^{\circ}_{H_2O,l} - \Delta G^{\circ}_{H_2O,g}$ ≈ -8.56 kJ/mol

Applying this correction gives the Gibbs free energy for the reaction with liquid water:

$\Delta G^{\circ}_{RWGS,H_2O(l)}$= $\Delta G^{\circ}_{RWGS,H_2O(g)}$ + ΔG°_{cond}

At 350 °C (623 K):

$\Delta G^{\circ}_{RWGS,H_2O(l)}$= +6.44 kJ/mol

At 300 °C (573 K):

$\Delta G^{\circ}_{RWGS,H_2O(l)}$= +8.54 kJ/mol

3. Equilibrium Constants

At 623 K:

$K_P(623\text{ K}) = \exp(-\frac{\Delta G^0(T)}{RT})$ ≈0.289

At 573 K:

$K_P(573\ \mathrm{K}) = \exp(-\frac{\Delta G^0(T)}{RT})$ ≈0.166

4. Equilibrium Conversion Calculation

Water in liquid phase ($a_{H_2O(l)} = 1$);

Initial partial pressure: $P_{CO_2} = 2\ bar$, $P_{H_2} = 3\ bar$

Reaction progress: ξ (mol, used in partial pressure fractions)

Equilibrium relation:

$$K_P = \frac{P_{CO}}{P_{CO_2} * P_{H_2}}$$

Solving for ξ gives:

350 °C (623 K): ξ ≈0.856 CO_2 conversion and CO yield: $\xi/2$≈0.428≈42.8%

300 °C (573 K): ξ ≈0.620 CO_2 conversion and CO yield: $\xi/2$≈0.310≈31.0%

Energy Consumption Analysis

Energy consumption analysis for CO_2 hydrogenation using Fe@C–Cu/Al_2O_3 activated by ACMF (80 mT, 350 kHz) or conventional heating at 350 °C:[27,48]

Under magnetocatalytic conditions (80 mT, 350 kHz), the Fe@C component in the Fe@C–Cu/Al_2O_3 catalyst absorbs approximately 6.4 W of power from the alternating magnetic field and converts it into thermal energy, corresponding to a total energy input of ~0.5 MJ over 2 h. Notably, the catalyst reaches its steady working temperature almost instantaneously. Under these conditions (43.5 mL reactor volume, 2 bar CO_2, 3 bar H_2, 2 h), a CO yield of 36% was achieved.

In contrast, with conventional heating the autoclave took 80 min to reach the target 350 °C, consuming approximately 1.2 MJ of electrical energy, as measured by a power meter. Maintaining the reactor at 350 °C for an additional 2 h consumed a further ~1.6 MJ. Despite this substantially higher energy input (~2.8 MJ in total), the catalytic performance remained poor, with only ~4.6% CO yield. (see Table below for a summary).

Thus, magnetically induced local heating enables significantly higher catalytic performance while requiring an order of magnitude lower energy input compared with conventional heating (0.5 MJ vs. 2.8 MJ; see below Table for a summary).

Estimation of heat generation under ACMF:

For 180 mg of Fe@C–Cu/Al_2O_3 catalyst containing 21.4 mg Fe, and a specific absorption rate (SAR) of ~300 W/gFe at 80 mT and 350 kHz, the Fe@C releases ~6.4 W of heat in the reactor. This corresponds to an energy input of ~0.5 MJ over a 2 h reaction period.

	Magnetic (80 mT, 350 kHz)	Conventional (350 °C)
Time to target T°C (h)	**0**	**1.33**
Reactor T°C	**130**	**350**
Reaction time (h)	**2**	**2**
Energy input to reactor (MJ)	**0.5**	**2.8**
CO Yield	**36**	**4.6**
Energy efficiency toward product formation (mmol MJ^{-1})	**2.5**	**0.058**

Table S1. ICP-OES analysis (measured under Ar) for Cu/Al_2O_3; ICNPs; Fe@C and Fe@C-Cu/Al_2O_3.

Sample Name	Al (wt%)	Cu (wt%)	Fe (wt%)	Atomic ratio		Theoretical atomic ratio	
				Cu/Al	Cu/Fe	Cu/Al	Cu/Fe
Cu/Al_2O_3	9.5	63.8	-	2.9	-	2	-
ICNPs	-	-	72.9	-	-	-	-
Fe@C	-	-	82.3	-	-	-	-
Fe@C-Cu/Al_2O_3	12.4	56.2	11.9	1.92	4.15	2	4.4

Table S2. Low-temperature (4 K) Mössbauer characterization of Fe@C and ICNPs: Fitting parameters and derived nanoparticle composition

Sample	Phases	δ (mm/s)	ΔEQ (mm/s)	μ_0H_{Hyp} (T)	Quantity (%)
Fe@C	Paramagnetic species	0.14	1.66	-	7.5
	Fe(0)	0.12	0.01	34.16	92.5
ICNPs	Paramagnetic species	0.39	1.19	-	3.5
	Fe(0)	0.09	0.00	34.44	16.1
	Fe_5C_2	0.31	-0.04	26.16	28.6
	Fe_2C	0.36	0.07	19.20	51.8

Table S3. N_2 physisorption characterization of Cu/Al_2O_3 and Fe@C-Cu/Al_2O_3

Sample Name	Surface Area (m²/g)	Pore Volume (cc/g)	Pore Diameter Dv(d) (nm)
Cu/Al_2O_3	106	0.146	3.63
Fe@C-Cu/Al_2O_3	88	0.124	3.02

Table S4. Experimental estimation of the surface temperature of ICNPs, Fe@C and Fe@C-Cu/Al_2O_3 under magnetic induction heating. Conditions: ICNPs (10 mg), Fe@C (10 mg), 17 wt% Fe@C-Cu/Al_2O_3 (180 mg), 0.5 mL solvent, under Ar atmosphere; f = 350 kHz, μ_0H = 72 mT, heating duration: 5 min.

**Solvent**	**Boiling point (°C)**	**Refluxing upon magnetic induction heating?**		
		ICNPs	Fe@C	Fe@C-Cu/Al_2O_3
Dodecane	216	Yes	Yes	Yes
Tetradecane	254	Yes	Yes	Yes
Hexadecane	287	Yes	Yes	Yes
Tetraethylene glycol	327	**Yes**	**Yes**	**No**

Table S5. MIC experiment with an additional heat-gun to keep the produced water in the gas phase. Conditions: Fe@C–Cu/Al_2O_3 (17 wt%, 180 mg), 5 bar (H_2:CO_2 = 3:2), 350 kHz, μ_0H = 80 mT for 2 h in a 43.5 mL reactor, heat gun at 120 °C.

Catalyst	Conversion %	Product yield %			
		CH_4	CO	C_2H_6	C_{3+}
Fe@C-Cu/Al_2O_3	25.7	0.87	23.3	0.41	1.16

Table S6. Comparison of the rWGS performances for Fe@C–Cu/Al_2O_3 and representative catalysts reported in the literature in recent years.

Catalyst	H_2/CO_2	Temp.℃	Press (bar)	Conv.%	Sel.%	Ref.
1Pt/Al_2O_3	1.0	500	1	33	100	Appl. Catal., A, 2012, 423–424, 100–107.
1Pt/TiO_2	1.0	500	1	38	100	Appl. Catal., A, 2012, 423–424, 100–107.
1Pt/TiO_2	1.0	400	1	21	95	Catal. Today, 2017, 281, 312–318
K80–0.3Pt/L	1.0	500	1	27	100	Appl. Catal., B, 2017, 216, 95–105.
Pt/10CeO_2–TiO_2	1.0	500	1	30	100	Environ. Technol., 2019, 182–192.
Cu/CeO_2	1.0	400	1	16	100	Appl. Catal., A, 2018, 562, 2836.
Ni/Ce–Zr–O	1.0	700	1	45	93	J. Hydrogen Energy, 2015, 40, 15985–15993.
NiO/CeO_2	1.0	700	1	40	100	Mater. Res. Bull., 2014, 53, 70–78.
7Ni/MgO	1.0	700	1	43	97	Environ. Chem. Eng., 2018, 6, 4945–4952.
20Ni/CeO_2	1.0	750	1	45	100	J. Rare Earths, 2008, 26, 6670.
1Mo/FAU	1.0	500	1	14.3	99	Appl. Catal., A, 2020, 592, 117415.
1Fe/FAU	1.0	500	1	5.9	98	Appl. Catal., A, 2020, 592, 117415.
1.1Cu–3.2Ni/Al_2O_3	1.0	600	1	37	93	Appl. Catal., B, 2020, 261, 118241.
2Rh–2.5Fe/TiO_2	1.0	270	20	9.16	28.4	Catal. Commun., 2010, 11, 901–906.
5Co–CeO_2	1.0	600	1	35	99.5	Catal. Today, 2018, 316, 155–161.
1K–3Cu–AlFe	1.0	550	1	38	100	ACS Catal., 2021, 11, 12609–12619.
Fe_3O_4	1.0	480	1	12.5	100	Catalysts, 2019, 9, 773.
MnO_2	1.0	850	1	44	100	Nanoscale, 2019, 11, 16677–16688.
MnO_2	1.0	850	1	51	100	Nanoscale, 2019, 11, 16677–16688.
In_2O_3–CeO_2	1.0	500	1	20	100	Catal. Today, 2016, 259, 402–408.
Ga_2O_3	1.0	600	1	19.1	100	Greenhouse Gases: Sci. Technol., 2014, 4, 140–144.
In_2O_3	1.0	600	1	29.6	100	Greenhouse Gases: Sci. Technol., 2014, 4, 140–144.
PtW/Al_2O_3	3.0	450	6.7	42	79	, J. Mater. Chem. A, 2021, 9, 15613–15617.
Pt/CeO_2-AA	3.0	450	1	34	100	Appl. Catal., B, 2021, 291, 120101.
CuSiO/CuO_x	3.0	500	1	17.8	100	Chem. Commun., 2019, 55, 4178–4181.
Cu/ZnO-SBA-15	3.0	400	1	29	100	Catal. Today, 2022, 402, 60–66.
3Mo/TiO_2-RNR	3.0	275	24	6.8	65.2	Green Chem., 2021, 23, 7259–7268.
Cu–In–Ni	3.0	580	1	50	100	Catal. Today, 2018, 316, 155–161.

Continued

Catalyst	H_2/CO_2	Temp.	Press (bar)	Conv.%	Sel.%	Ref.
$1Mo/TiO_2$-DT51D	3.0	275	24	5.2	74.6	Green Chem., 2021, 23, 7259–7268.
$ZnO–Al_2O_3$	3.0	700	1	67	100	Appl. Catal., A, 2001, 211, 81–90.
$ZnO–Cr_2O_3$	3.0	700	1	67	100	J. Chem. Eng., 2000, 17, 719–722.
WC	3.0	350	20	24.3	88	J. CO_2 Util., 2020, 35, 38–46.
Na-WC	3.0	350	20	13.6	98.1	J. CO_2 Util., 2020, 35, 38–46.
K-WC	3.0	350	20	20.3	100	J. CO_2 Util., 2020, 35, 38–46.
Mo_2C/N–C	3.0	560	1	40	100	J. Energy Chem., 2020, 50, 37–43.
VC	3.0	600	1	44	100	Appl. Catal., B, 2020, 267, 118719.
CuK/C	3.0	260	20	17.5	100	ACS Catal. 2024, 14, 9188−9197
a-Mo_2C	3.0	500	1	50	100	Science 384, 540–546 (2024)
a-Mo_2C	3.0	600	1	60	100	Science 384, 540–546 (2024)
$Pt_1Fe_1@SiO_2$	3.0	450	1	44	99.5	J. Am. Chem. Soc. 2024, 146, 27555−27562
$(Ni0.2Mg_{0.2}Cu_{0.2}Zn_{0.2}Co_{0.2})Fe_2O_4$	3.0	400	1	41.4	99.6	Applied Catalysis B: Environment and Energy 349 (2024) 123845
Cu_9Co_1/Al_2O_3	3.0	400	1	32.5	99.4	ACS Catal. 2024, 14, 7020−7031
H_xMoO_y	3.0	500	1	36.40	100	Angew. Chem. Int. Ed. 2024, 63, e202411761
FFe B	3.0	400	1	17	84	ChemCatChem 2024, 16, e202301398
Pt-Mo_2N-0.2	3.0	350	1	15	93	Adv. Funct. Mater. 2024, 2413043
MoOx	3.0	500	1	43.1	90.2	Nature Communications | (2024)15:3100
$[(THF)KFe(OtBu)_2]/Al_2O_3$−500	3.0	400	30	22.5	100	ACS Catal. 2024, 14, 2418−2428
$Pt/BaZr_{0.9}Y_{0.1}O_{3-\delta}$, Pt/BZY_{10})	3.0	500	1	38	100	Catal. Sci. Technol., 2024, 14, 6076–6084
Y_2O_3/Cu	3.0	600	1	45.6	99.9	Catal. Sci. Technol., 2024, 14, 3483–3492
Mon+Mo_2C/OCNT	3.0	600	1	37	100	ACS Catal. 2024, 14, 10939−10950
CoPd/Co@C	3.0	476	1	71.1	99	ACS Catal. 2025, 15, 9489−9502
FeCo@FeCoOx@C	3.0	376	1	74.2	100	ACS Catal. 2025, 15, 10663−10673
FeCo@FeCoOx@C	3.0	190	1	54.4	99.5	ACS Catal. 2025, 15, 10663−10673
FeCo@FeCoOx@C	3.0	169	1	33.6	99.7	ACS Catal. 2025, 15, 10663−10673
**Fe@C-Cu/Al_2O_3**	**1.5**	**125**	**5**	**53**	**90**	**This work**
**Fe@C-Cu/Al_2O_3**	**3.0**	**125**	**5**	**66.6**	**93**	**This work**

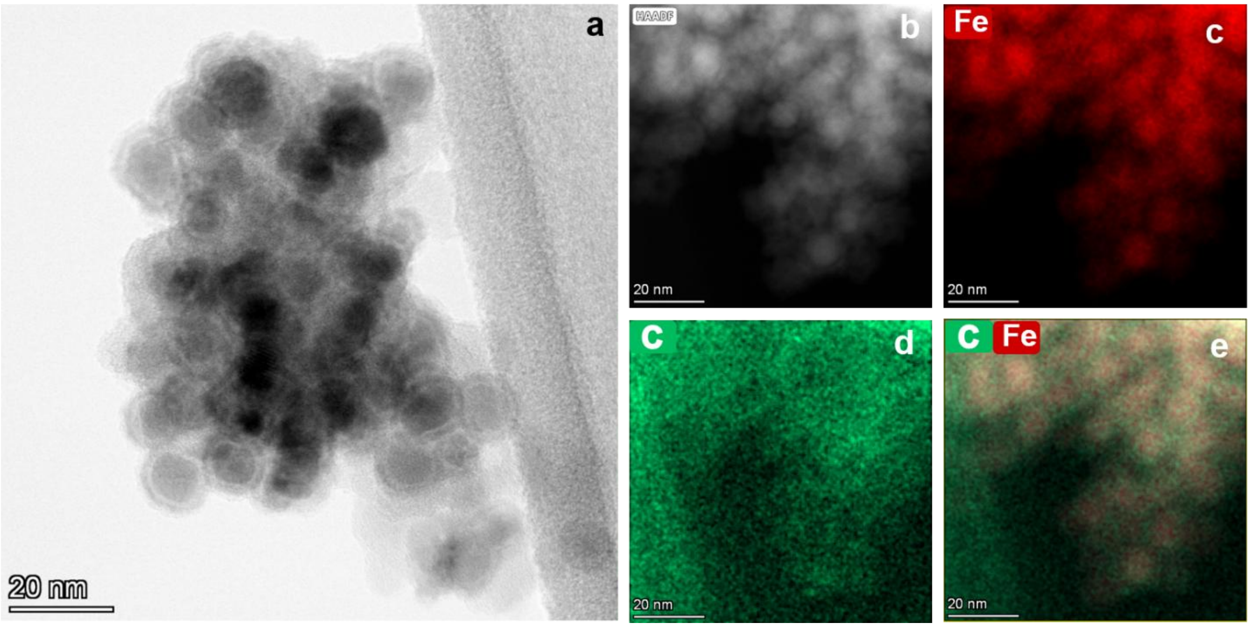

Figure S1. Characterization of ICNPs by a) HRTEM and b-e) HAADF-STEM-EDX.

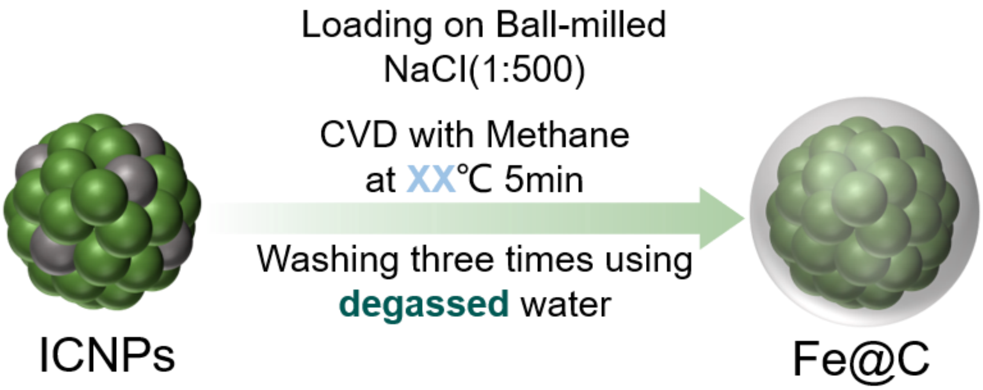

Figure S2. Schematic illustration of the synthesis process of Fe@C.

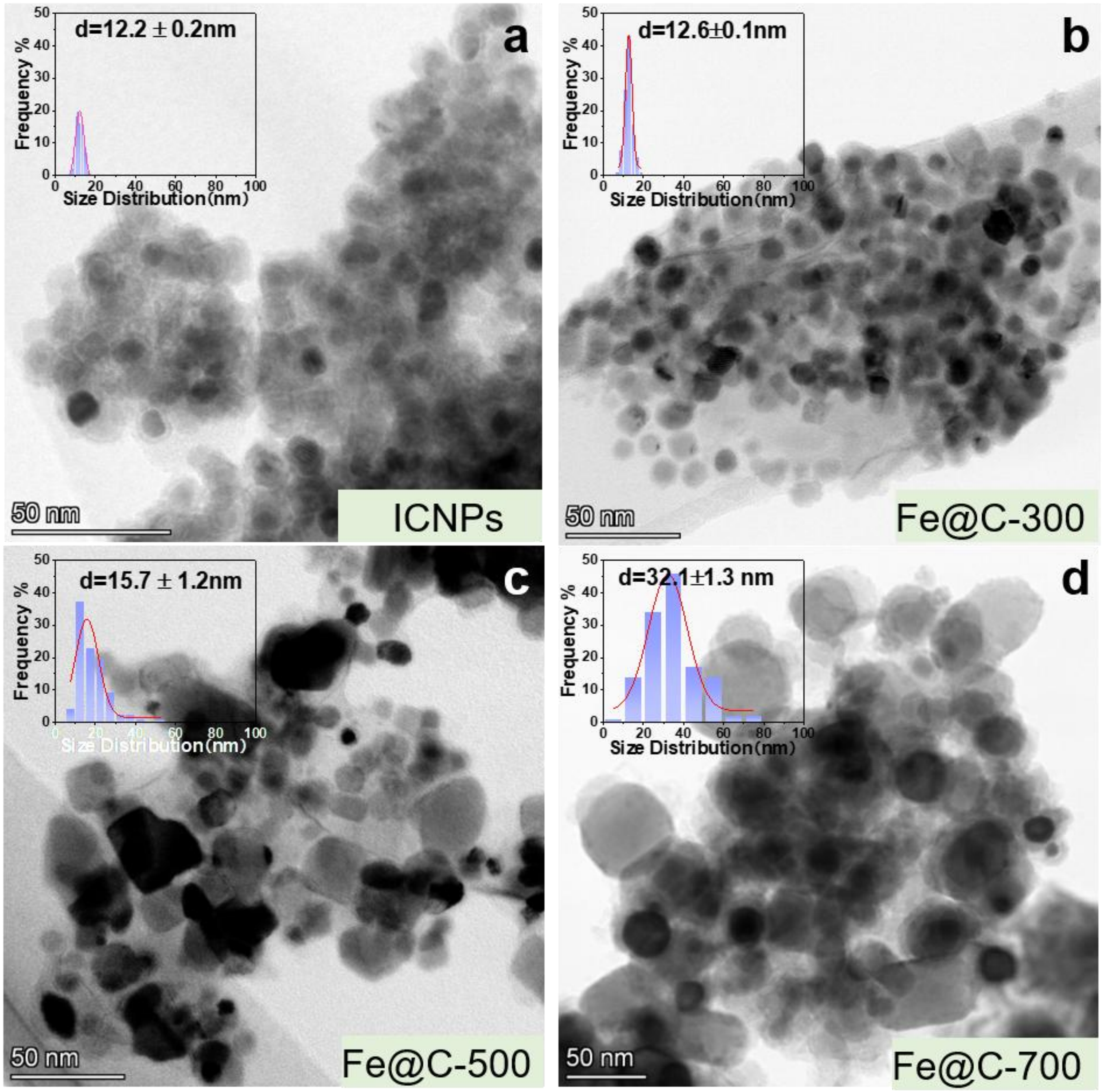

Figure S3. Characterization by TEM of Fe@C NPs prepared at different CVD temperatures. a, starting ICNPs; b, Fe@C-300 (CVD at 300 degree for 5 min); c, Fe@C-500 (CVD at 500 degree for 5 min); and d, Fe@C-700 (CVD at 700 degree for 5 min).

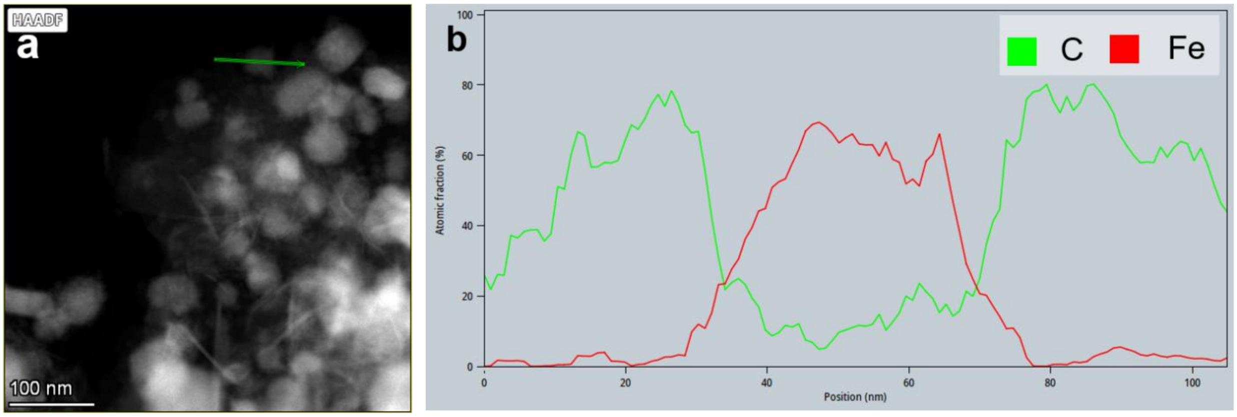

Figure S4. Structural and compositional analysis of the selected Fe@C (Fe@C-700). a) HAADF-STEM image of Fe@C; b) EDX line scan profile showing Fe and C distribution across the nanoparticle.

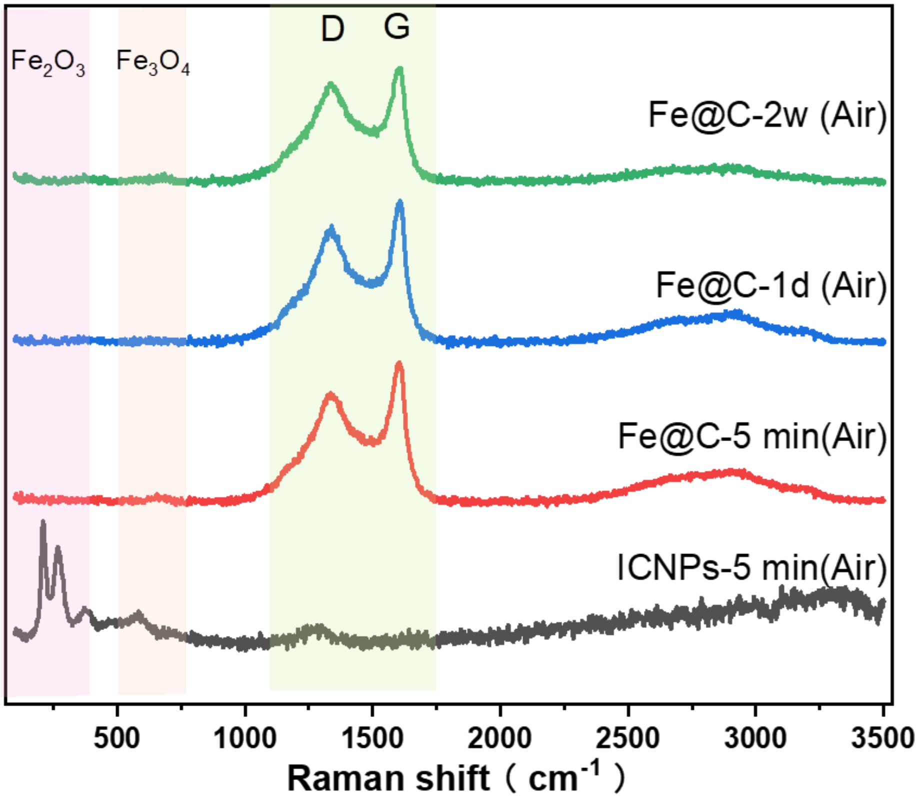

Figure S5. Raman spectra of Fe@C and ICNPs after different Air exposure times. 5 min =5 minutes, 1d = 1 day, 2w = 2 weeks.

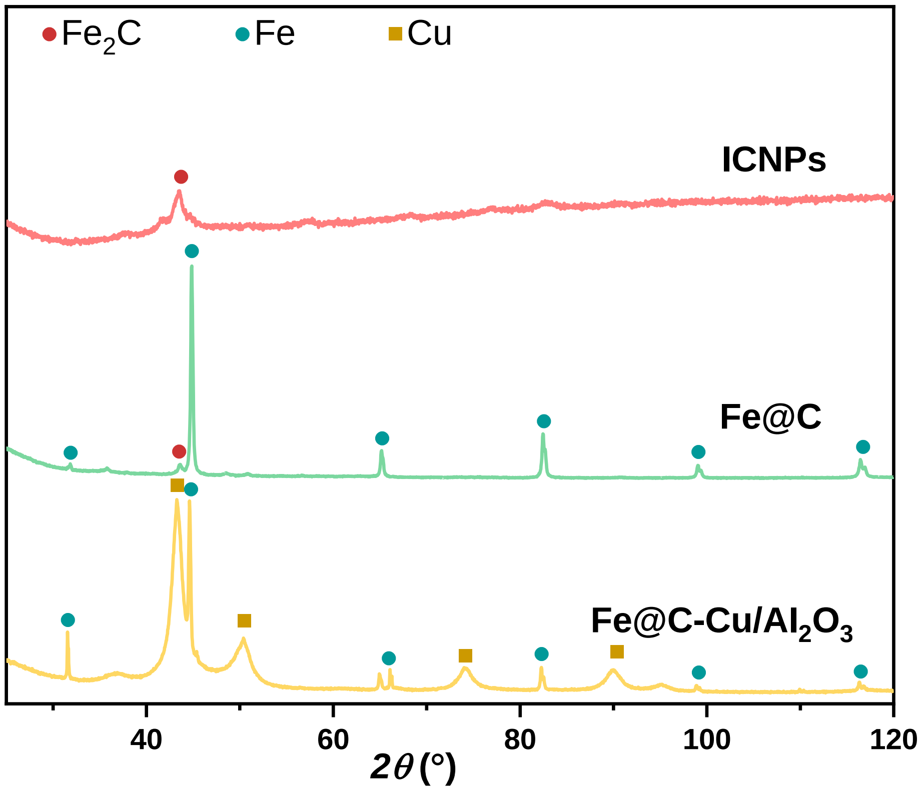

Figure S6. XRD patterns of ICNPs, Fe@C, and Fe@C–Cu/Al_2O_3 (Fe_2C: PDF# 36-1249; Fe: PDF# 06-0696; Cu: PDF# 85-1326)

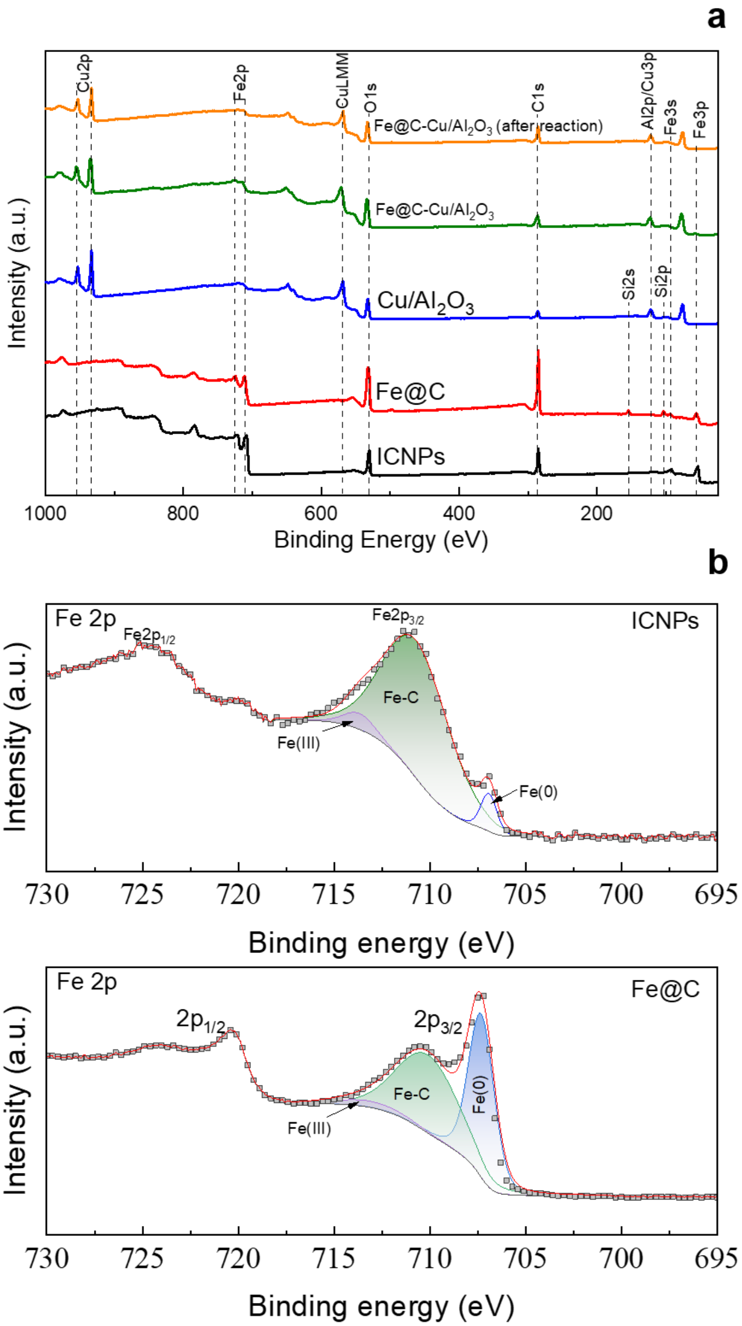

Figure S7. a) XPS survey spectra of Fe@C, ICNPs, and Fe@C-Cu/Al_2O_3 samples; b) High-resolution Fe 2p XPS spectra of ICNPs and Fe@C. Attribution based on ref[49].

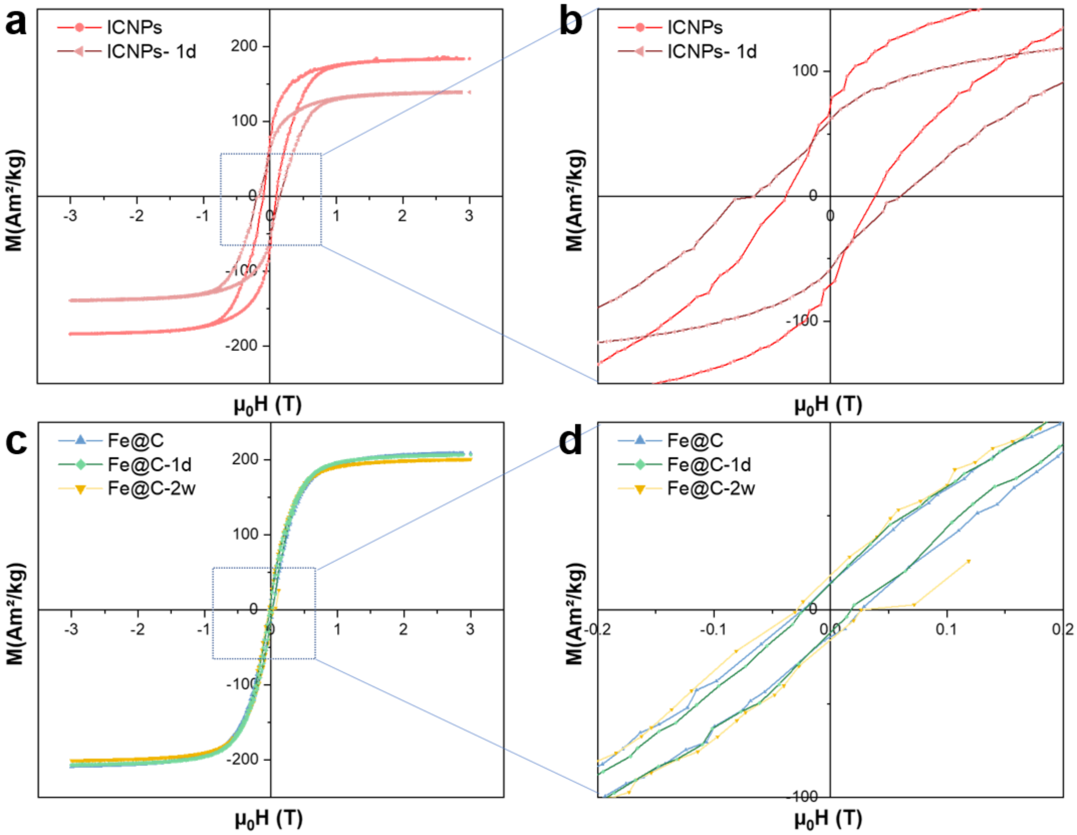

Figure S8. VSM (5 K) characterization of ICNPs (a-b) and Fe@C (c-d) after different Air exposure times. 1d = 1 day; 2w = 2 weeks.

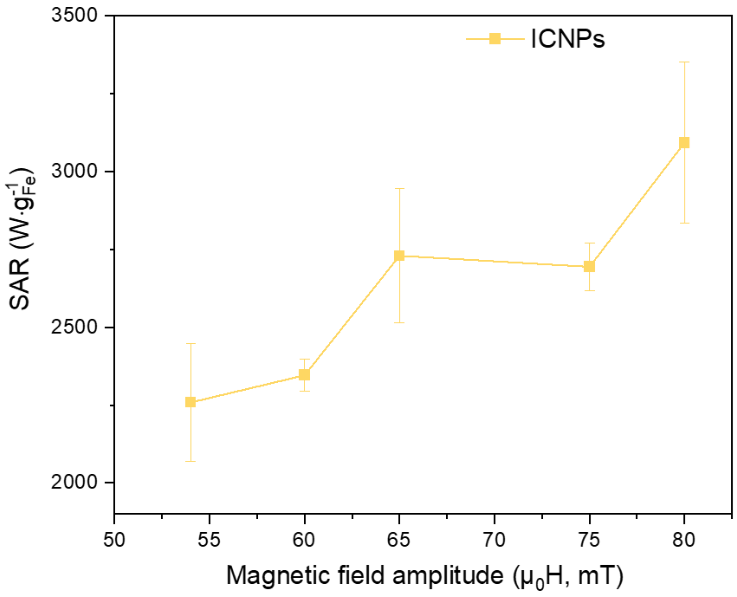

Figure S9. SAR measurements for ICNPs at 350 kHz.

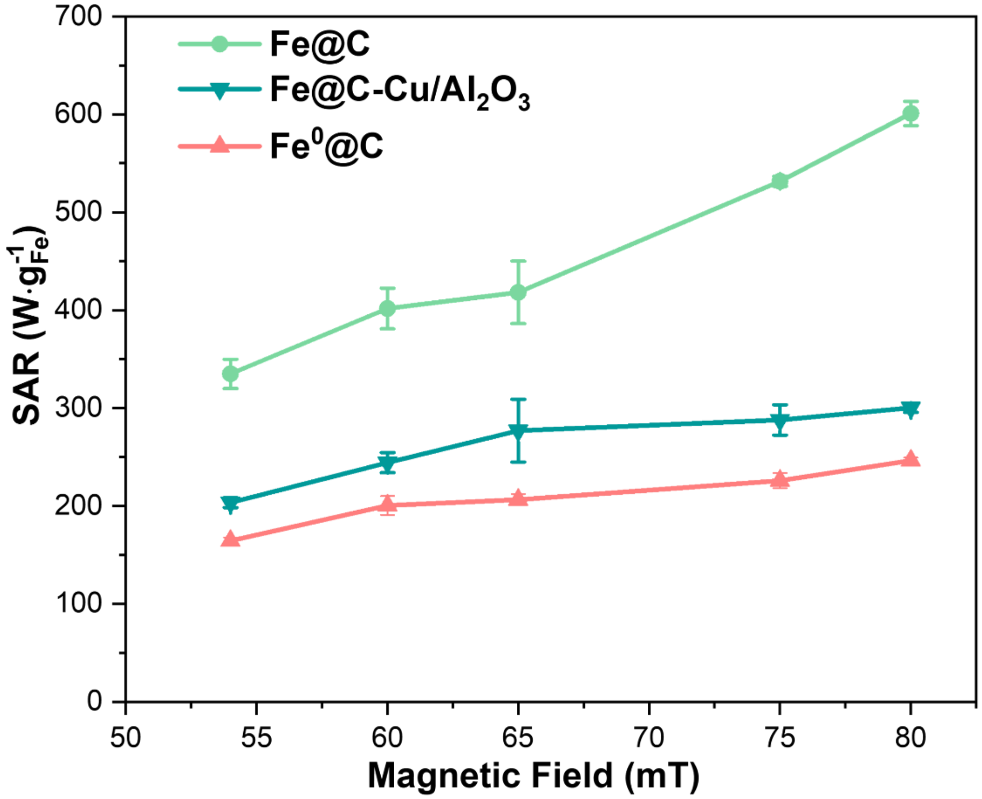

Figure S10. SAR measurements for Fe@C, Fe@C-Cu/Al_2O_3 and Fe^0@C at 350 kHz.

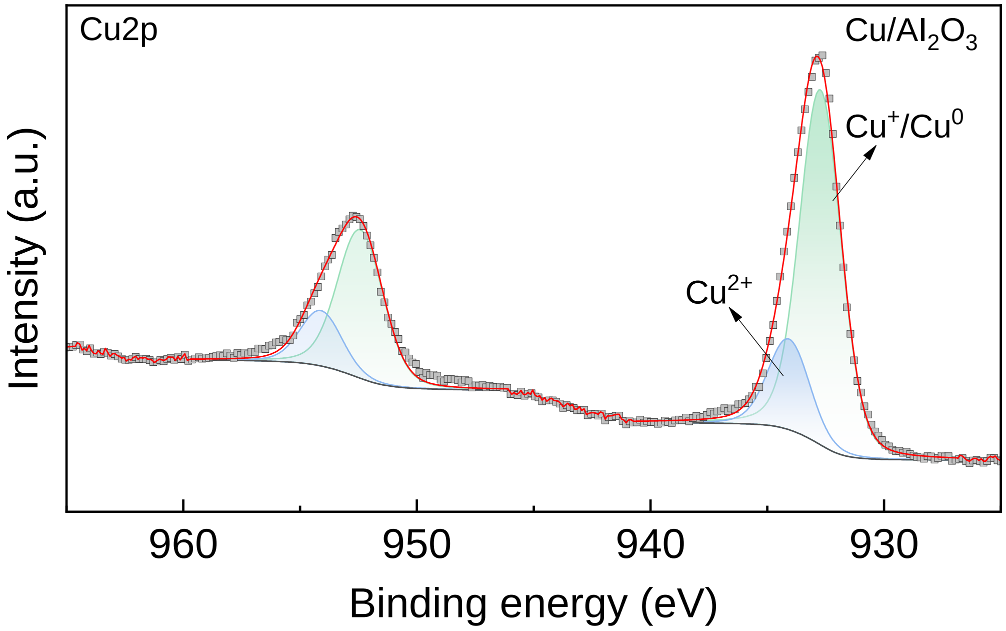

Figure S11. High-resolution Cu 2p XPS spectrum of Cu/Al_2O_3.

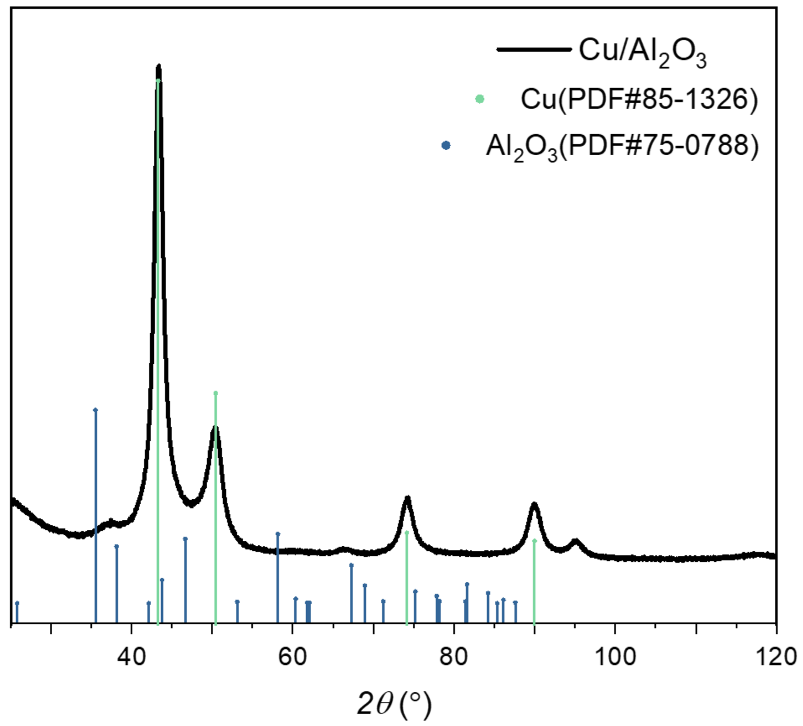

Figure S12. XRD analysis for Cu/Al_2O_3.

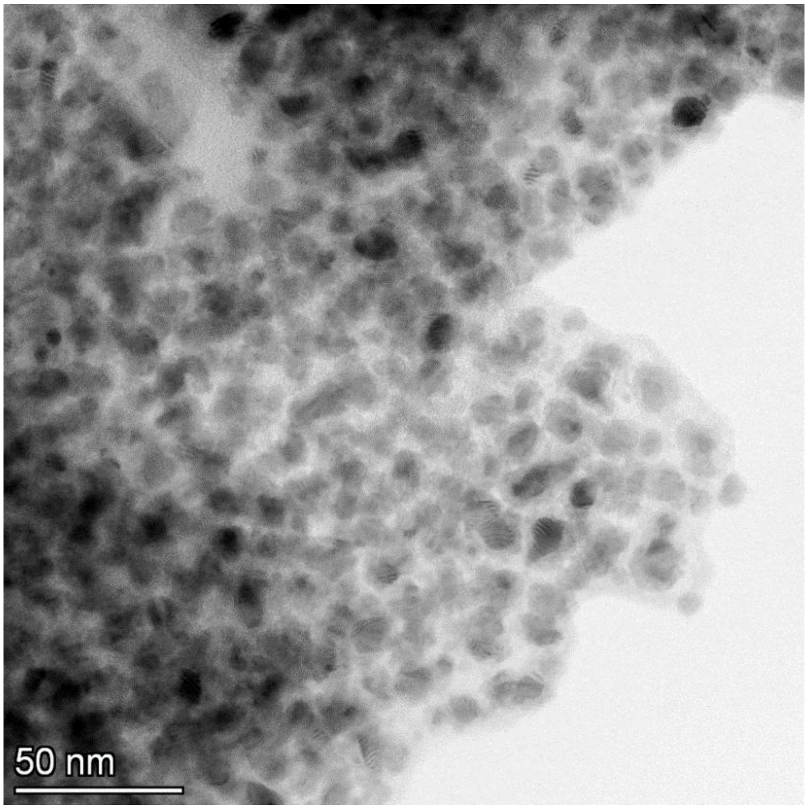

Figure S13. TEM image of Cu/Al_2O_3.

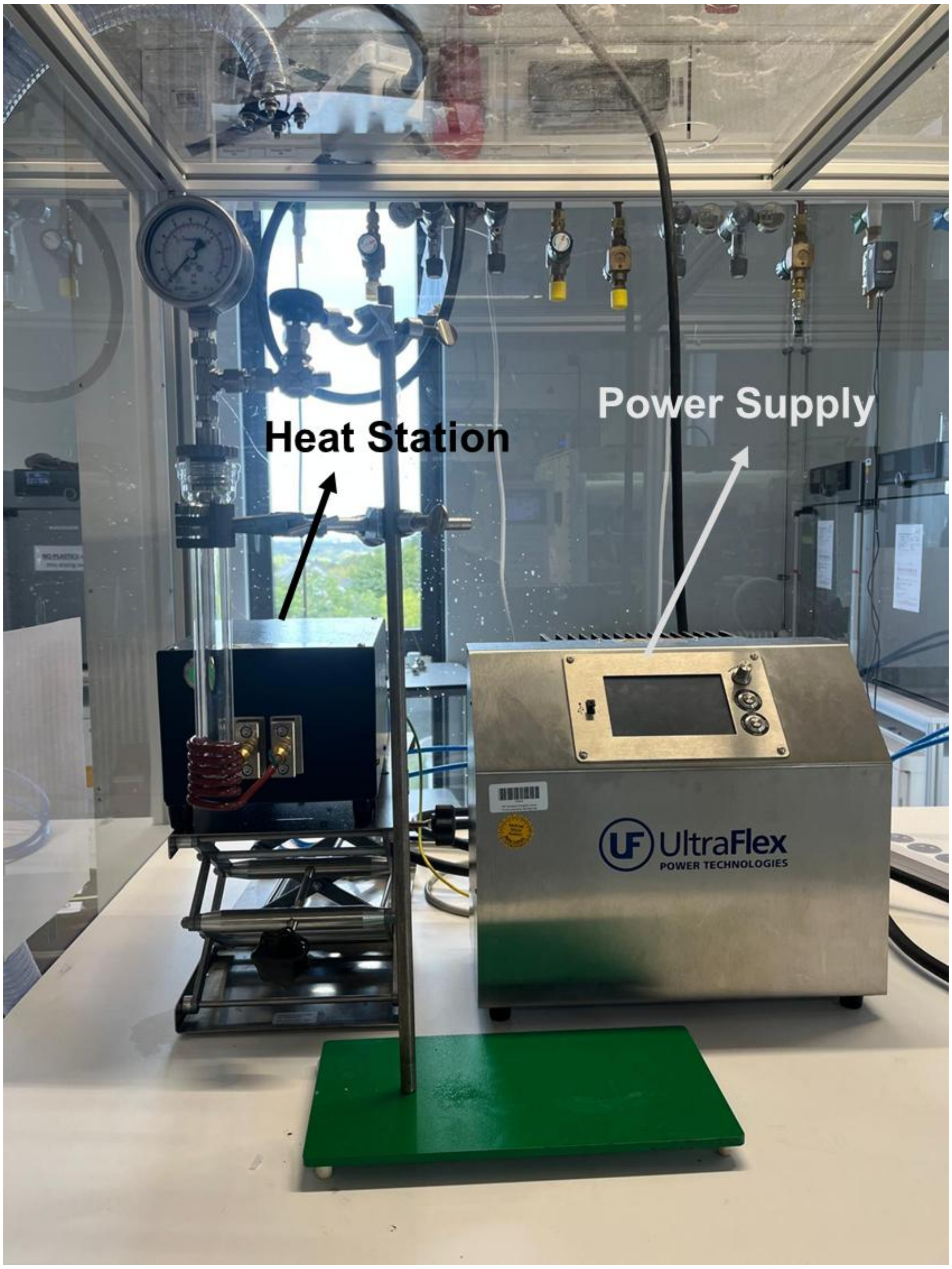

Figure S14. Picture of the magnetically induced catalysis set-up.

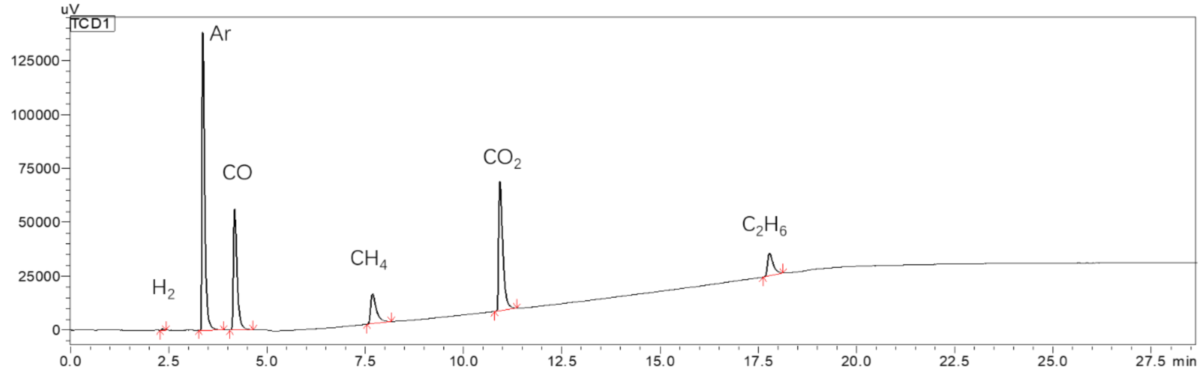

Figure S15. Typical gas chromatogram (GC-TCD) obtained from a certified standard gas mixture.

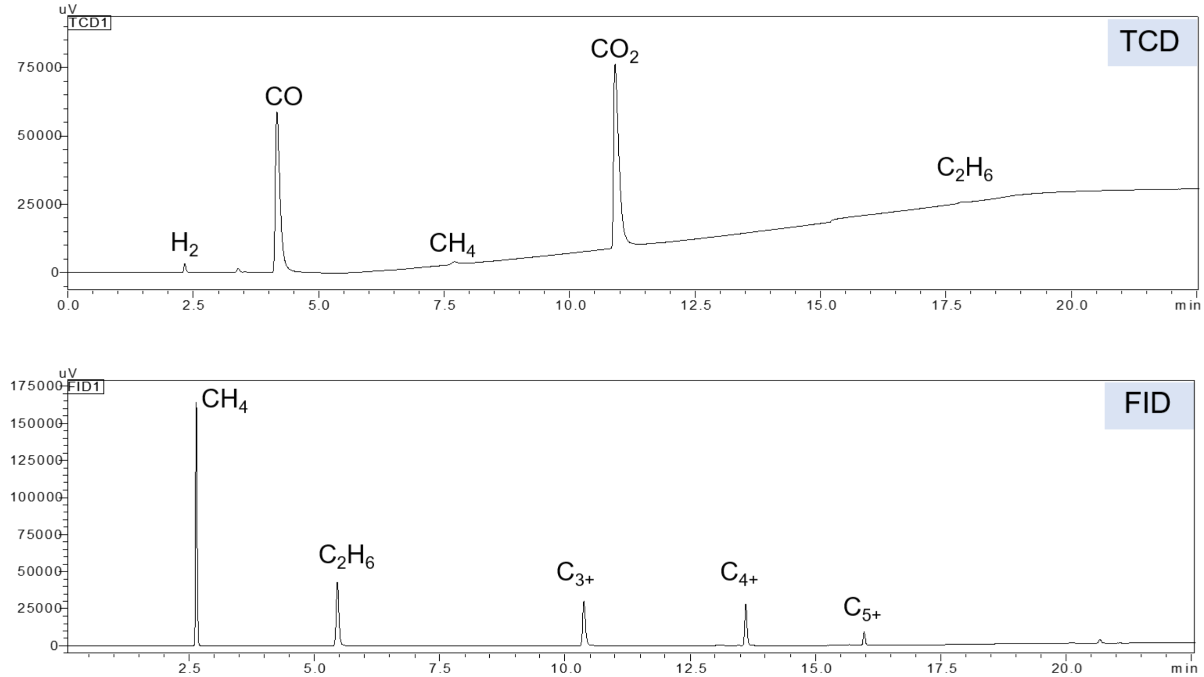

Figure S16. Example of GC-TCD and GC-FID results of CO_2 hydrogenation. Reaction conditions: Magnetic induction heating with Fe@C-Cu/Al_2O_3 (17 wt%, 180 mg), 5 bar (H_2:CO_2 = 3:2), 350 kHz, μ_0H = 80 mT for 2 h in a 43.5 mL Fisher-Porter bottle.

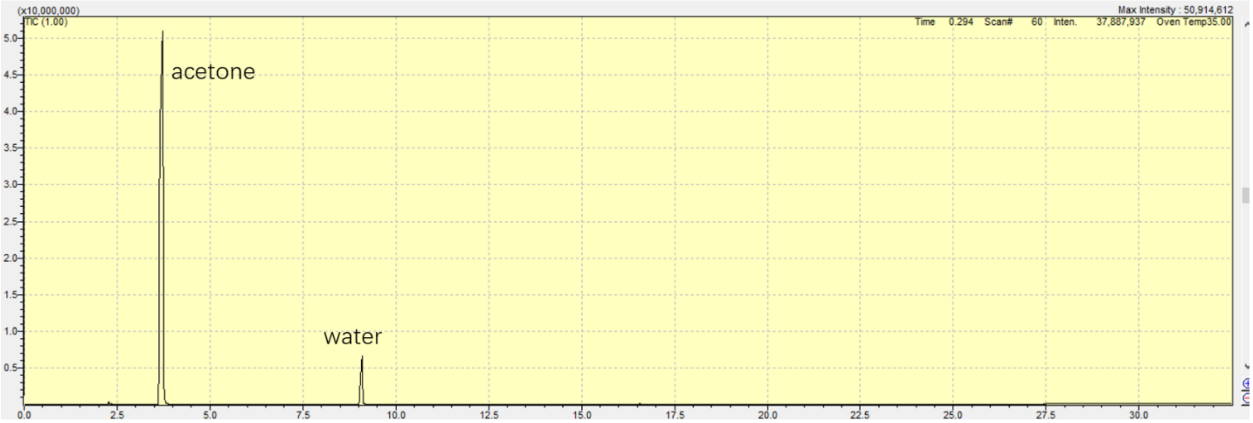

Figure S17. Representative example of the GC-MS analysis of potential liquid products. Acetone was used as the solvent to wash the Fisher-Porter bottle and collect potential products.

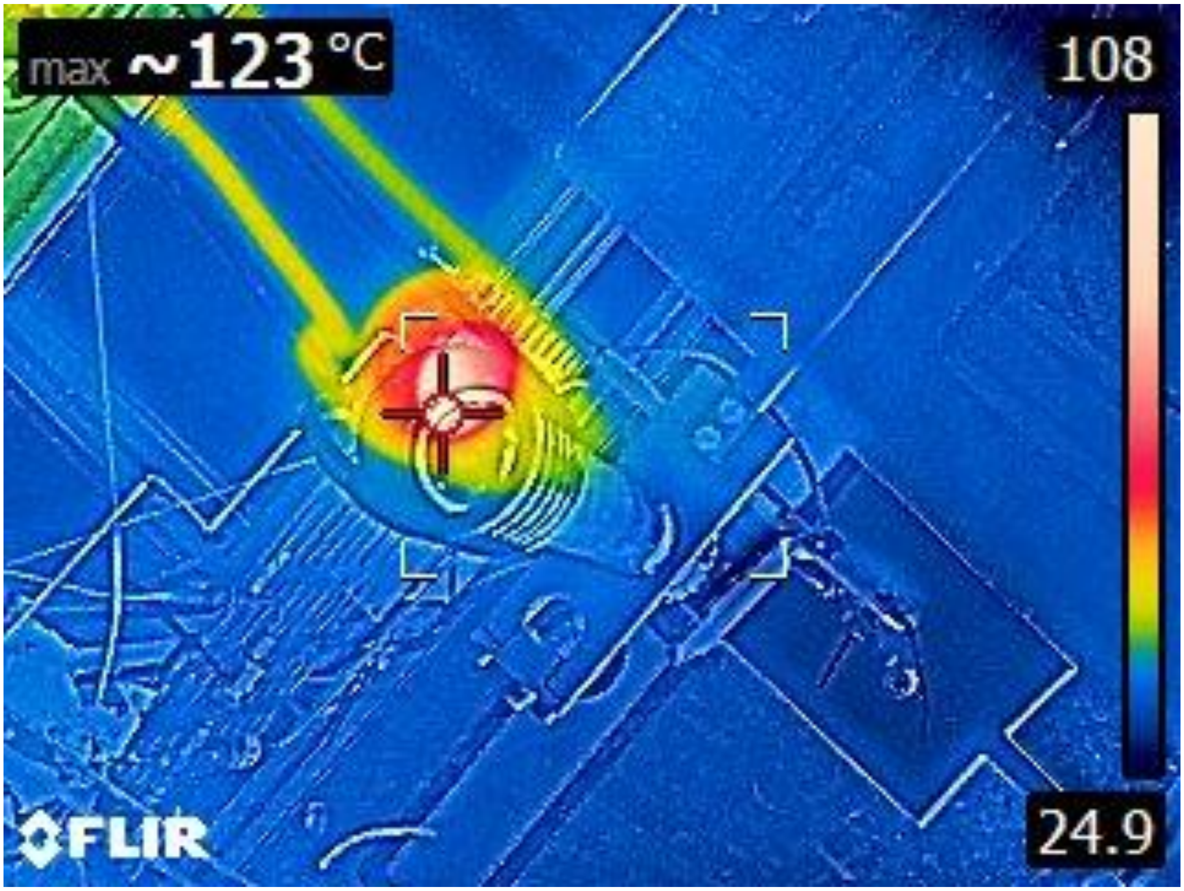

Figure S18. Infrared (IR) image of the reactor (from the bottom) under reaction conditions (80 mT, 350 kHz).

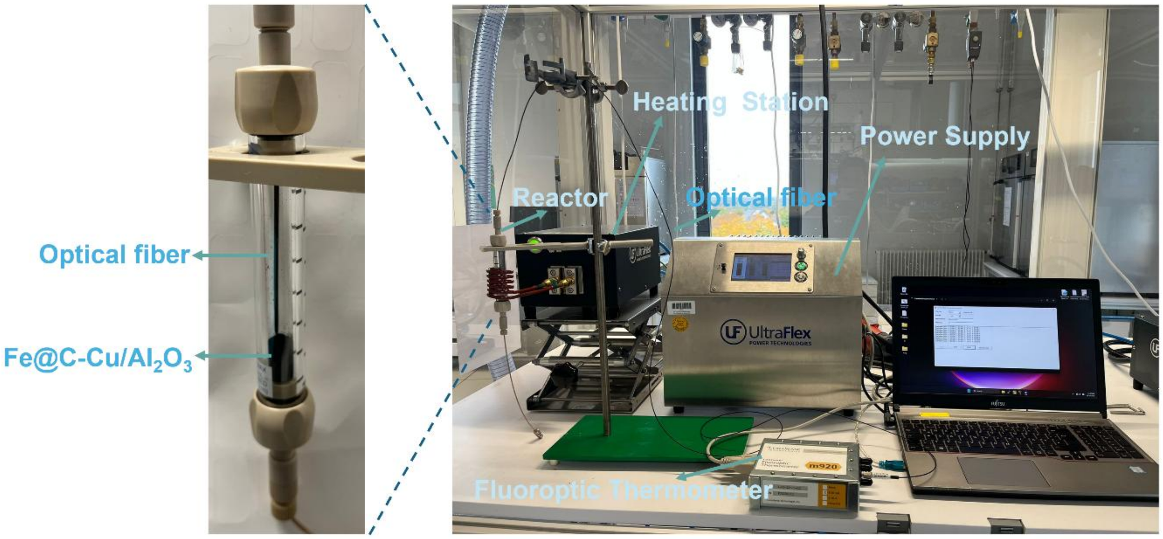

Figure S19. Experimental setup for the determination of the catalyst bed temperature using a fiber optic temperature sensor.

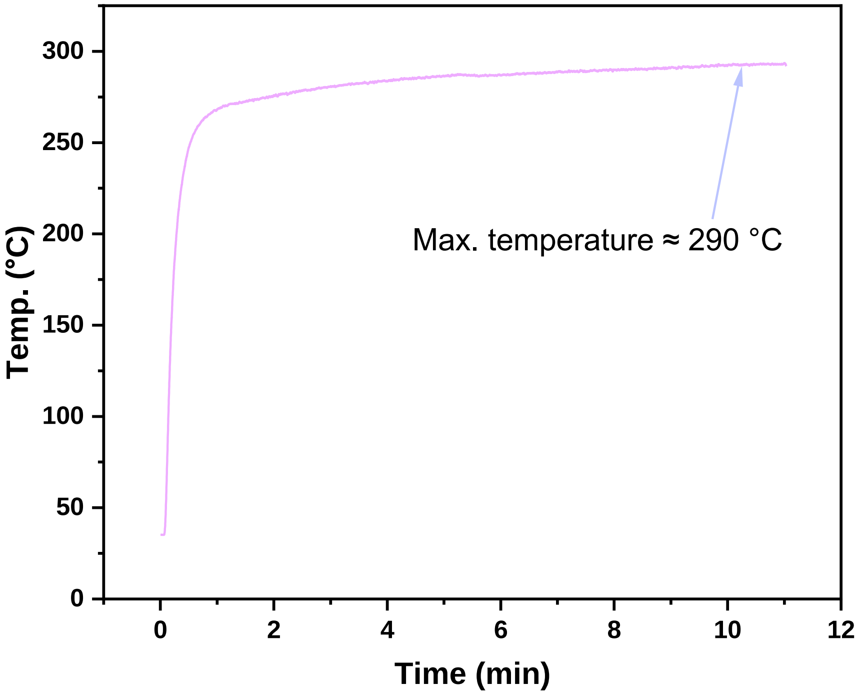

Figure S20. Monitoring of the temperature of a bed of Fe@C-Cu/Al_2O_3 catalyst heated by magnetic induction measured by a fiber optic temperature sensor. Conditions: 180 mg catalyst, Ar atmosphere; f = 350 kHz, μ_0H = 72 mT; heating time, 10 min.

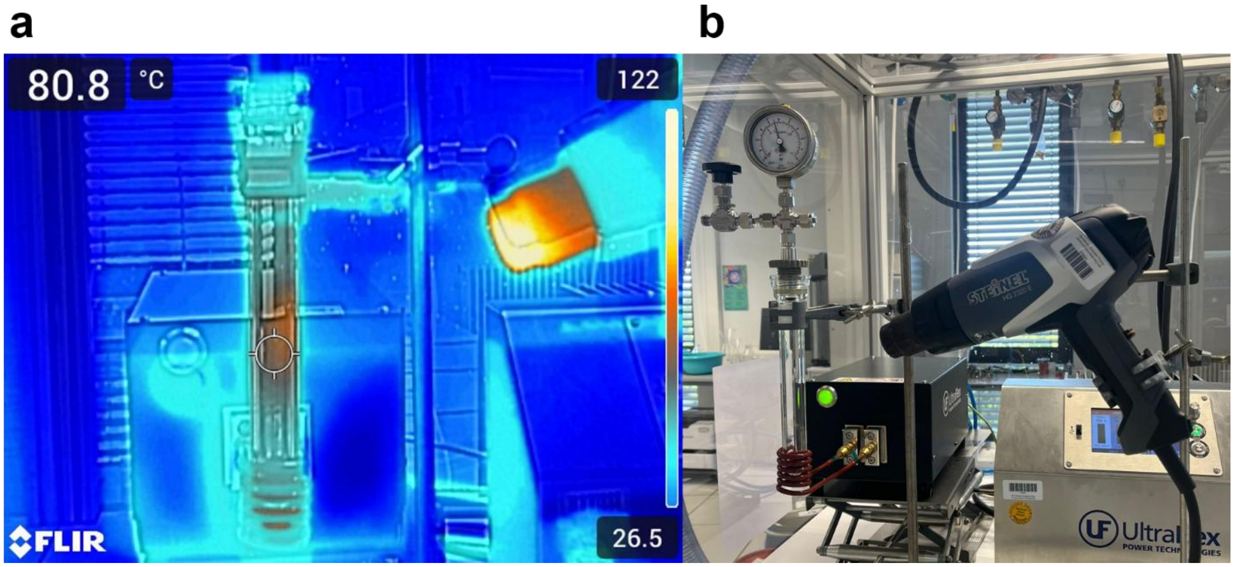

Figure S21. IR image (a) and photograph (b) of the magnetically induced catalytic hydrogenation of CO_2 with Fe@C-Cu/Al_2O_3 using a heat gun to heat the Fisher-Porter bottle walls and prevent water condensation. The heat gun was set to 120 °C, and the distance between the heat gun and the Fisher–Porter bottle was approximately 15 cm.

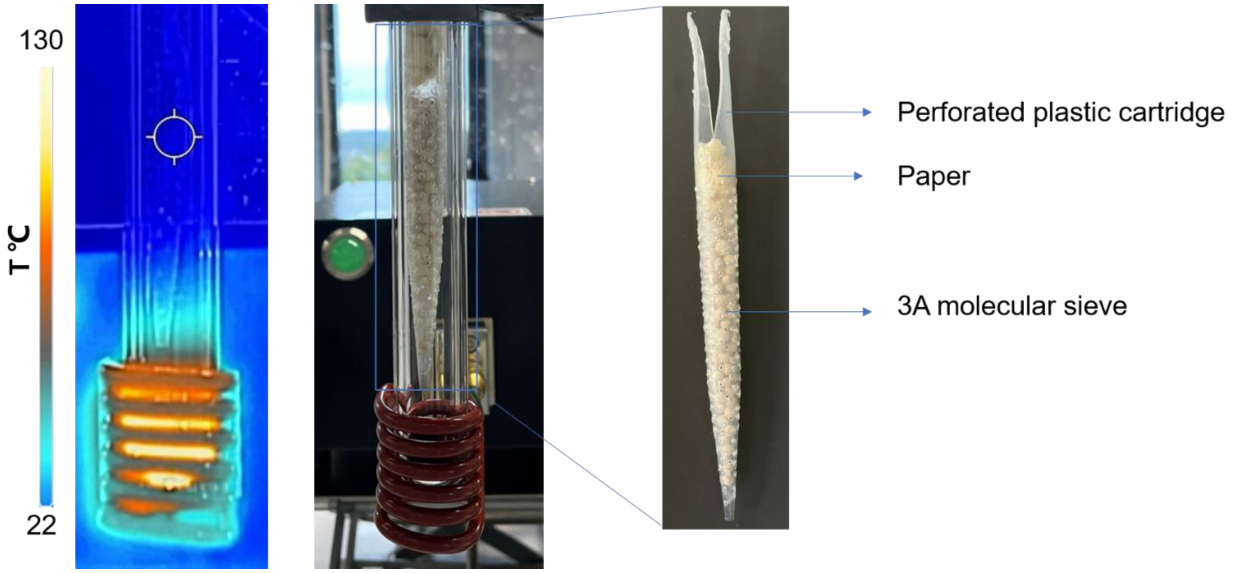

Figure S22. Photograph of the Fisher–Porter bottle reactor used for magnetically induced CO_2 hydrogenation. Approximately 2 g of 3 Å molecular sieves were added as a drying agent (pre-dried at 500 °C for 24 h and stored in a glovebox prior to use). The 3 Å molecular sieves do not adsorb CO_2; their maximum adsorption capacity is ~400 mg for 2 g

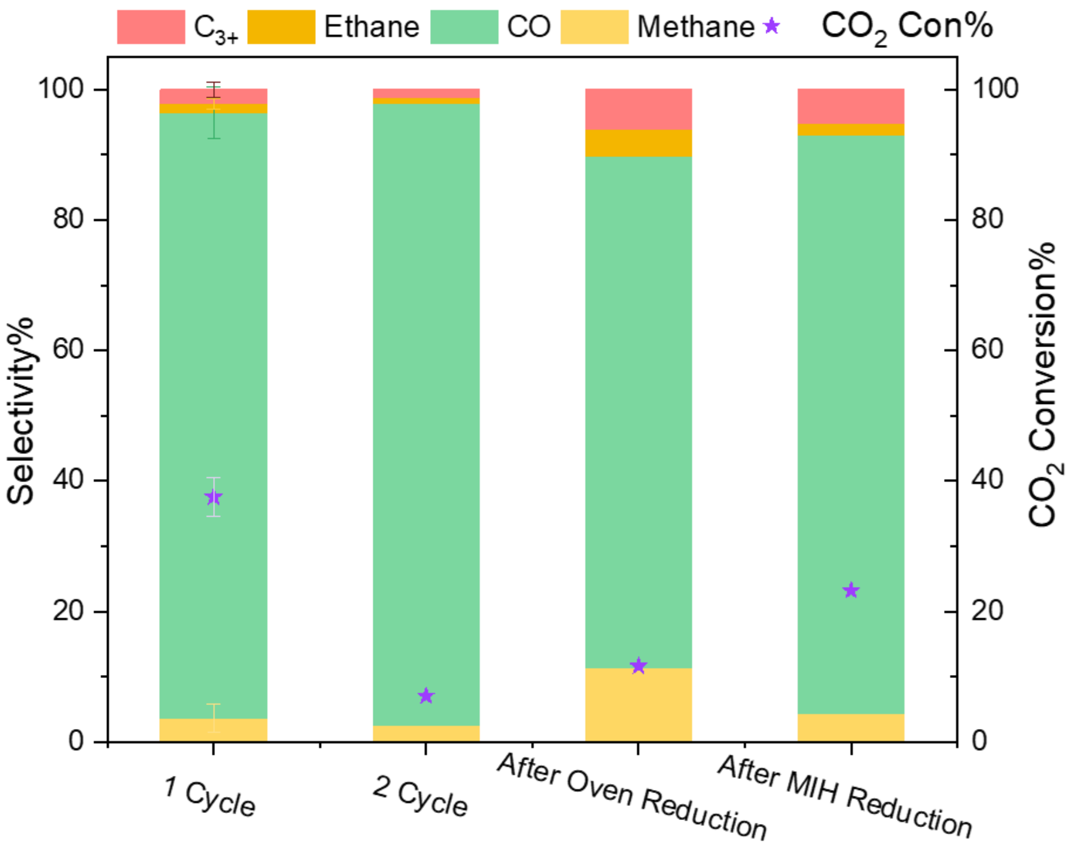

Figure S23. Recycling experiments using Fe@C-Cu/Al_2O_3 in magnetically induced rWGS. Reaction conditions: Fe@C-Cu/Al_2O_3 (180 mg, 17 wt%), 5 bar total pressure (H_2:CO_2 = 3:2), 350 kHz, 2 h, μ_0H = 80 mT, 43.5 mL sealed bottle. Oven reduction condition: heated to 300 °C at a rate of 1 °C·min^{-1} under a continuous H_2 flow (60 mL·min^{-1}) for 5 h. Magnetic induction heating (MIH) reduction condition: 5 bar H_2, 350 kHz, 1 h, μ_0H = 80 mT, 43.5 mL sealed bottle.

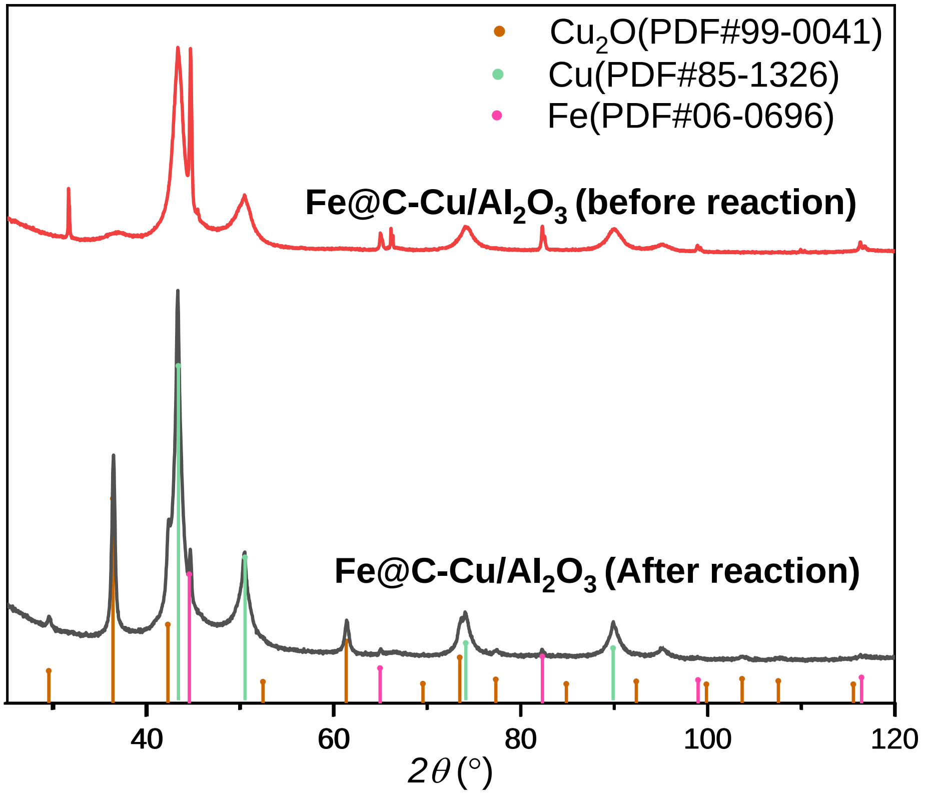

Figure S24. XRD analysis of Fe@C-Cu/Al_2O_3 after reaction under standard conditions.

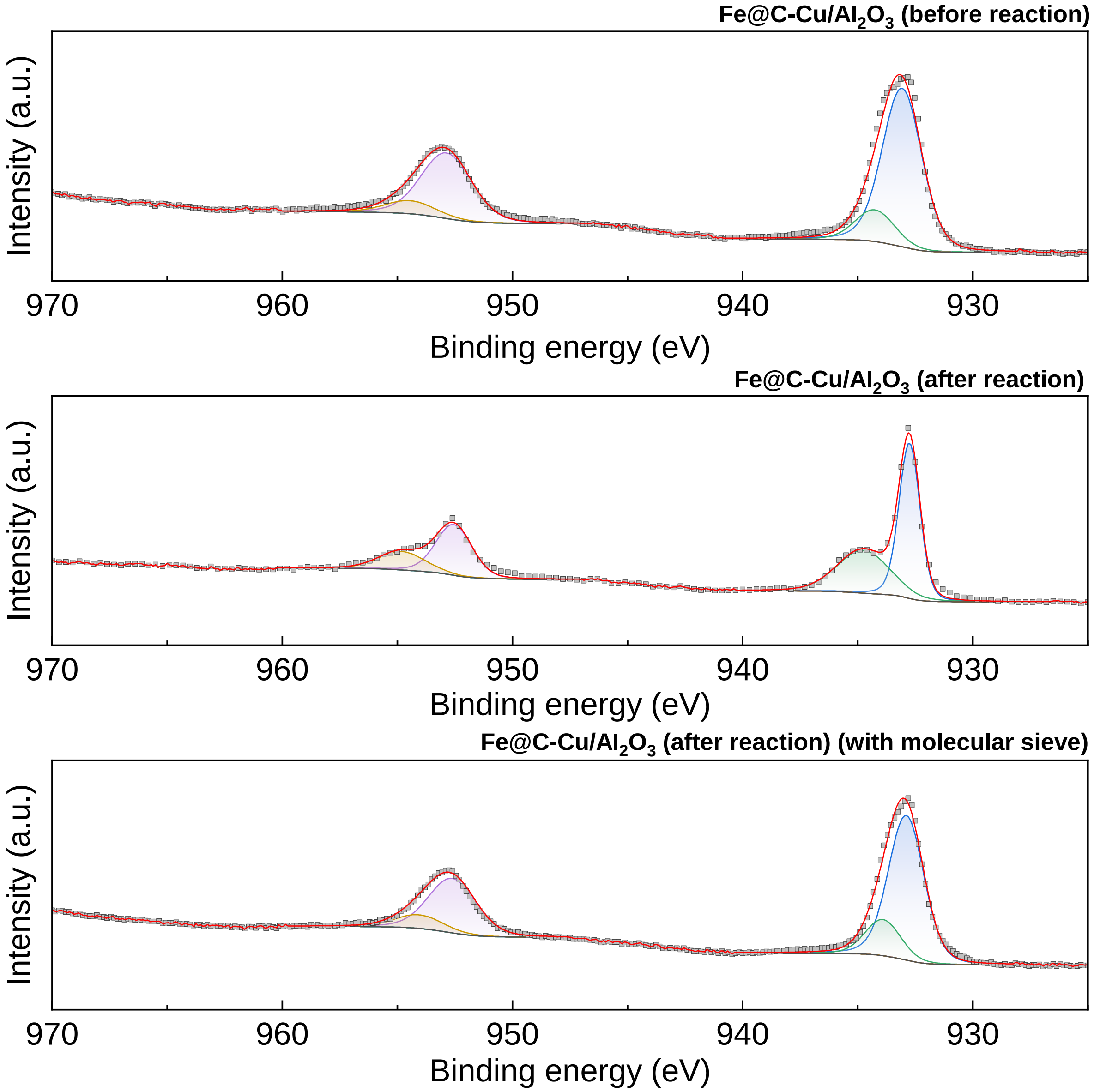

Figure S25. XPS characterization (Cu 2p) of Fe@C-Cu/Al_2O_3 before and after catalysis.

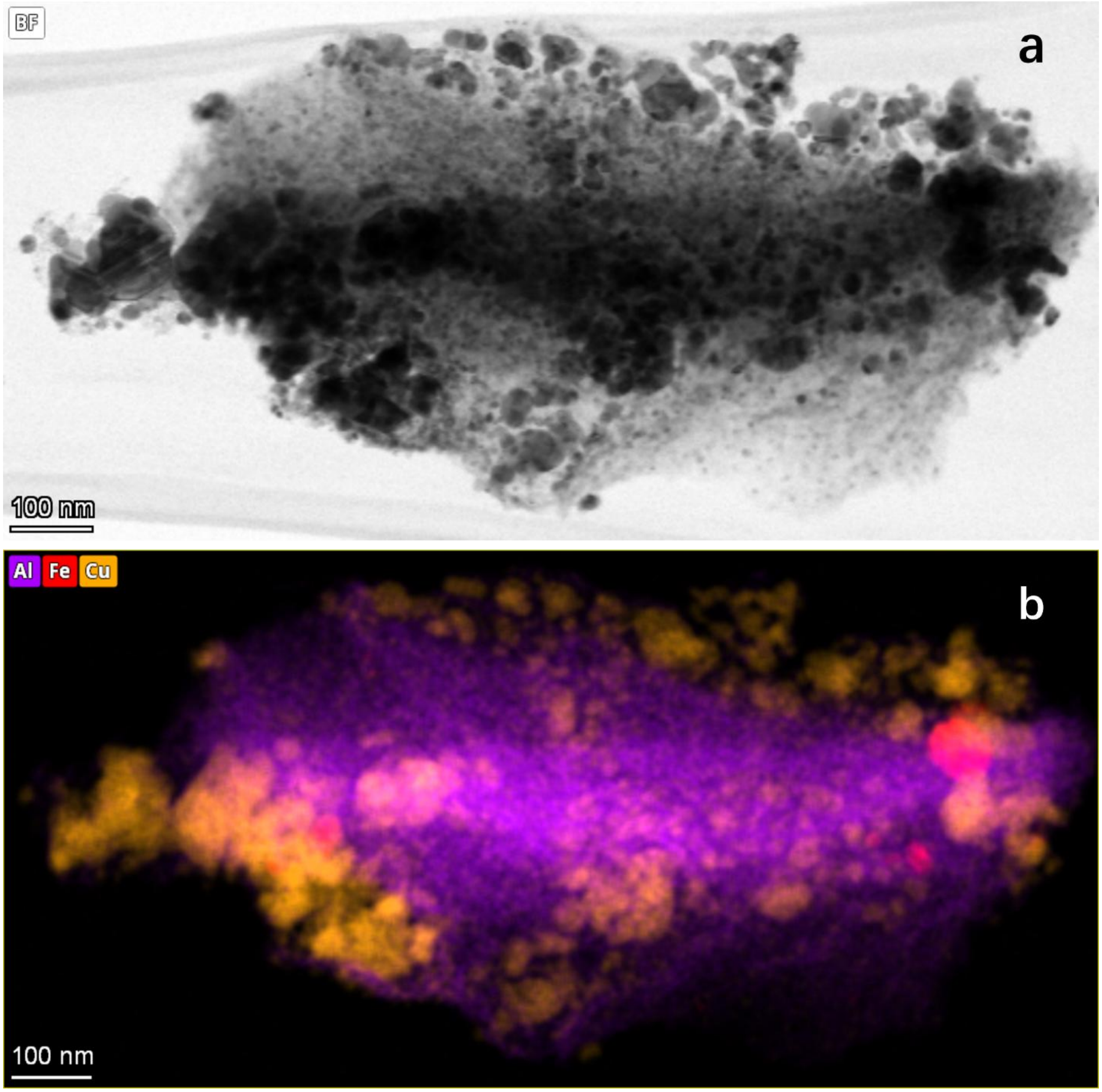

Figure S26. Electron microscopy characterization of the Fe@C-Cu/Al_2O_3 catalyst after reaction without molecular sieve. a) TEM; b) EDX elemental mapping.

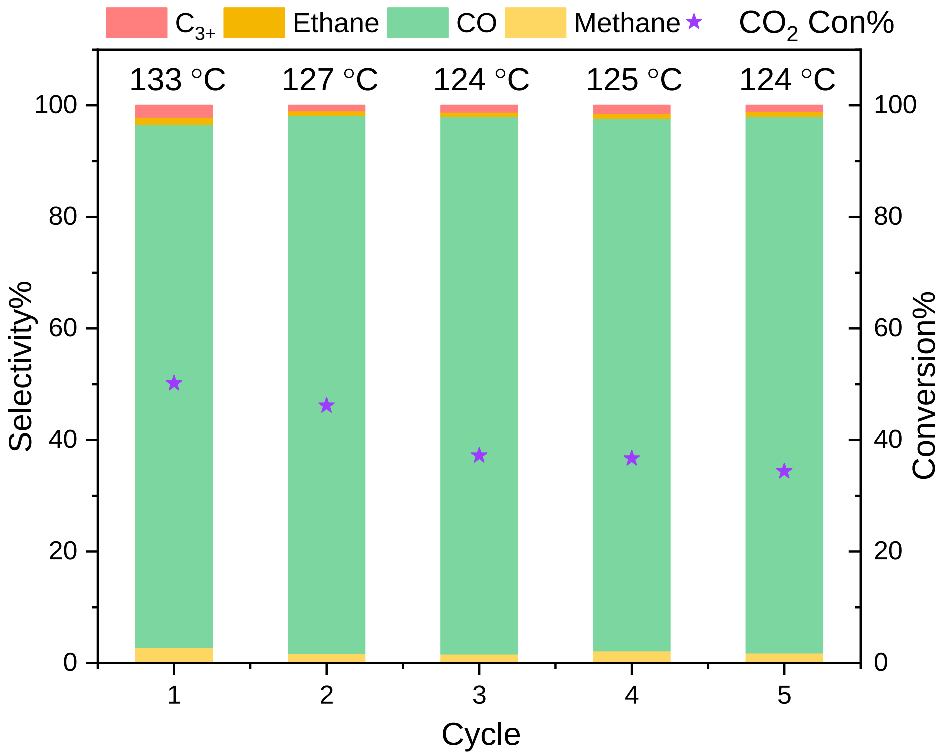

Figure S27. Recycling experiments with Fe@C-Cu/Al_2O_3 in CO_2 hydrogenation under magnetic induction heating in the presence of molecular sieves. Reaction conditions: Fe@C-Cu/Al_2O_3 (180 mg, 17 wt%), 5 bar total pressure (H_2:CO_2 = 3:2), 350 kHz, μ_0H = 80 mT, 43.5 mL sealed bottle, 1 h.

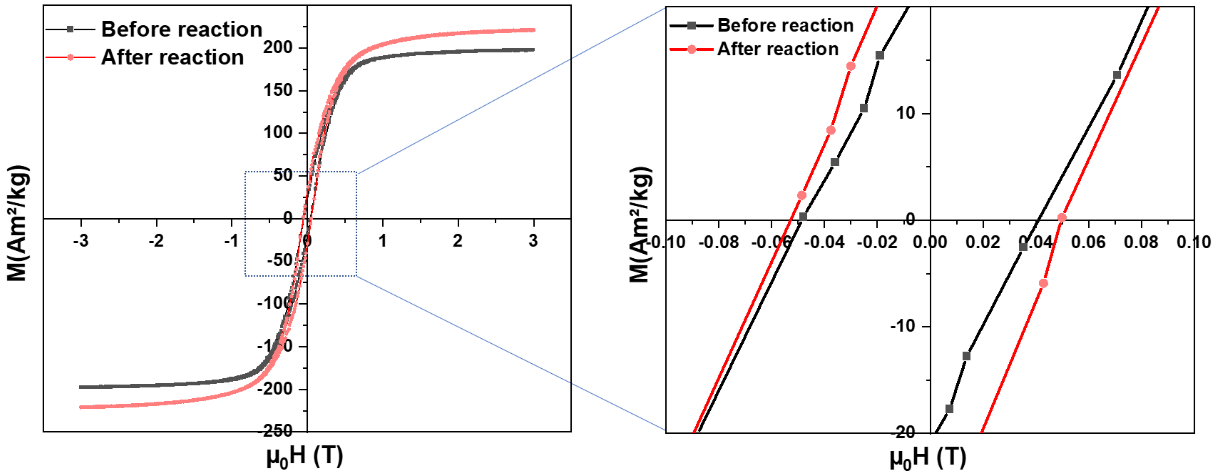

Figure S28. VSM (5K) analysis of Fe@C-Cu/Al_2O_3 after recycling experiments.

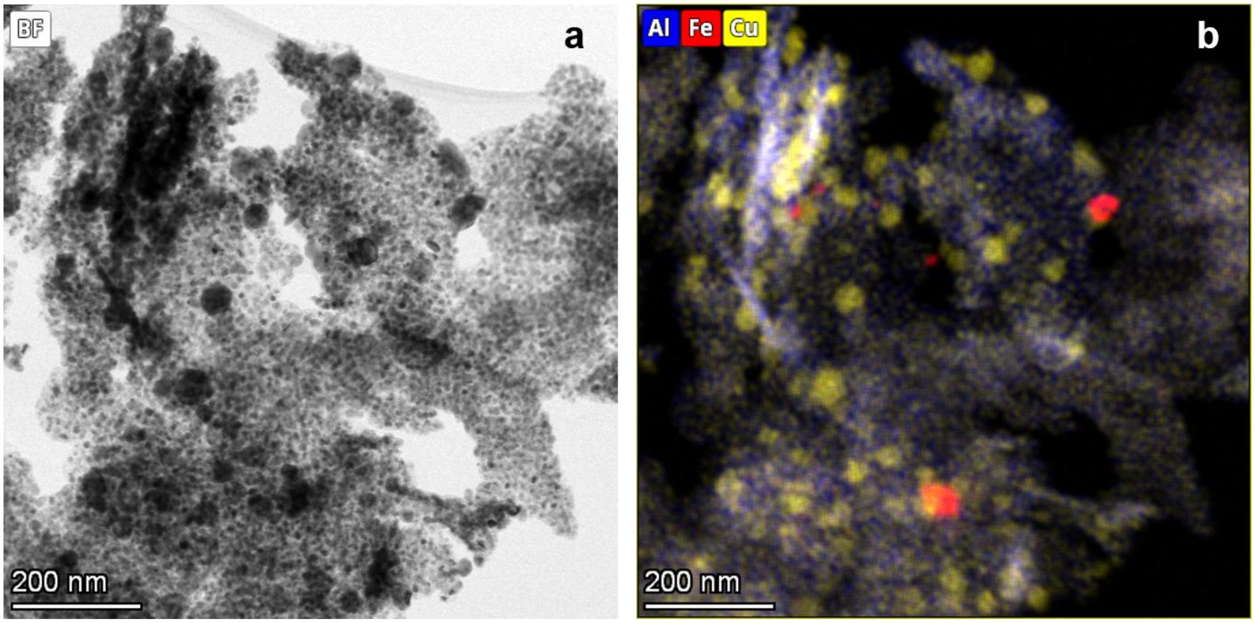

Figure S29. Electron microscopy characterization of the Fe@C-Cu/Al_2O_3 catalyst after recycling experiments. a) TEM; b) EDX elemental mapping.

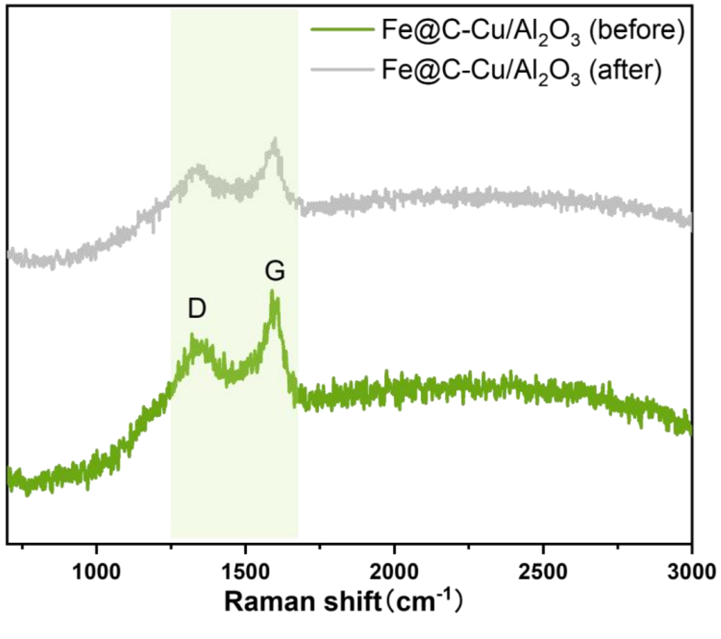

Figure S30. Raman characterization of Fe@C-Cu/Al_2O_3 before and after catalysis.

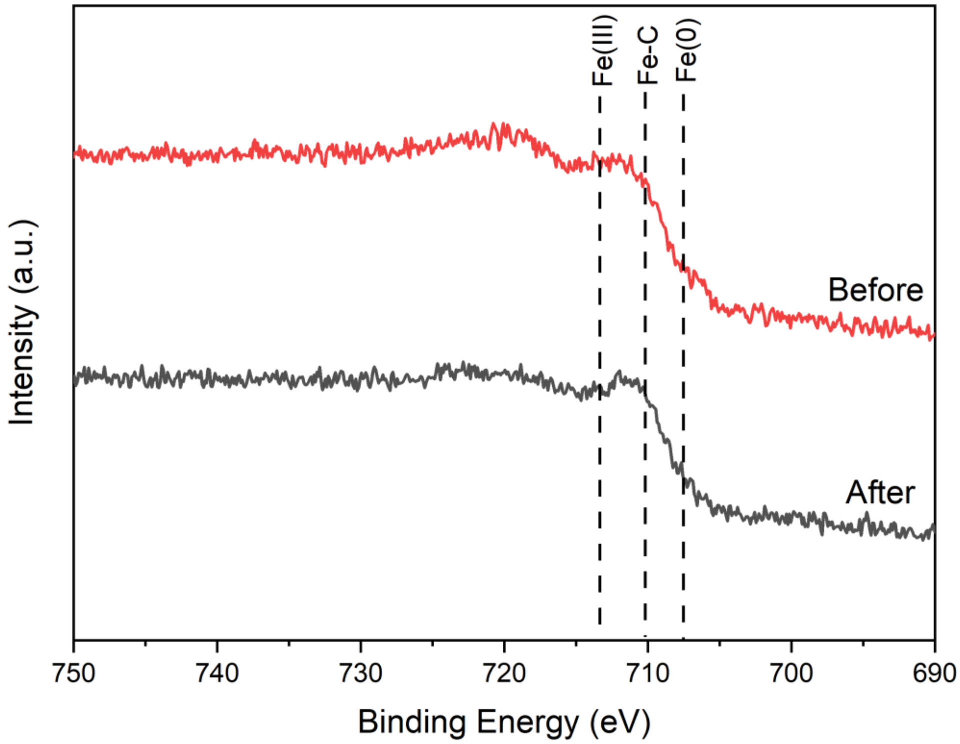

Figure S31. XPS characterization (Fe 2p) of Fe@C-Cu/Al_2O_3 before and after catalysis.

References and Notes

[1] Y. Choi, G. D. Sim, U. Jung, Y. Park, M. H. Youn, D. H. Chun, G. B. Rhim, K. Y. Kim, K. Y. Koo, "Copper catalysts for CO_2 hydrogenation to CO through reverse water–gas shift reaction for e-fuel production: Fundamentals, recent advances, and prospects" *Chem. Eng. J.* **2024**, *492*, 152283.

[2] L. Barberis, C. I. Versteeg, J. D. Meeldijk, J. A. Stewart, B. D. Vandegehuchte, P. E. de Jongh, "K and Na Promotion Enables High-Pressure Low-Temperature Reverse Water Gas Shift over Copper-Based Catalysts" *ACS Catal.* **2024**, *14*, 9188–9197.

[3] Z. Liu, F. Zhang, N. Rui, X. Li, L. Lin, L. E. Betancourt, D. Su, W. Xu, J. Cen, K. Attenkofer, H. Idriss, J. A. Rodriguez, S. D. Senanayake, "Highly Active Ceria-Supported Ru Catalyst for the Dry Reforming of Methane: In Situ Identification of $Ru^{\delta+}$–Ce^{3+} Interactions for Enhanced Conversion" *ACS Catal.* **2019**, *9*, 3349–3359.

[4] H. Lv, X. Dong, R. Li, C. Zeng, X. Zhang, Y. Song, H. Liu, J. Shao, N. Ta, Q. Zhao, Q. Fu, J. Xiao, G. Wang, X. Bao, "Super-dry reforming of methane using a tandem electro-thermocatalytic system" *Nat. Chem.* **2025**, 1–8.

[5] G. Garcia, E. Arriola, W.-H. Chen, M. D. De Luna, "A comprehensive review of hydrogen production from methanol thermochemical conversion for sustainability" *Energy* **2021**, *217*, 119384.

[6] R. Zhang, X. Liu, N. Song, J. He, Z. Cen, C. Li, M. Wang, H. Tang, W. Liu, X. Ren, D. Ma, "Magnetic Induction Heating-Driven Rapid Cold Start of Ammonia Decomposition for Hydrogen Production" *J. Am. Chem. Soc.* **2024**, *146*, 28635–28641.

[7] A. D. Sudipta De Adrian Ramirez, and Jorge Gascon, "Advances in the Design of Heterogeneous Catalysts and Thermocatalytic Processes for CO_2 Utilization" *ACS Catal.* **2020**, *10*, 14147−14185.

[8] J. Ye, N. Dimitratos, L. M. Rossi, N. Thonemann, A. M. Beale, R. Wojcieszak, "Hydrogenation of CO_2 for sustainable fuel and chemical production" *Science* **2025**, *387*, eadn9388.

[9] C. Jia, J. Gao, Y. Dai, J. Zhang, Y. Yang, "The thermodynamics analysis and experimental validation for complicated systems in CO_2 hydrogenation process" *J. Energy Chem.* **2016**, *25*, 1027–1037.

[10] A. Bordet, W. Leitner, B. Chaudret, "Magnetically Induced Catalysis: Definition, Advances, and Potential" *Angew. Chem. Int. Ed.* **2025**, *64*, e202424151.

[11] M. Cai, Z. Wu, Z. Li, L. Wang, W. Sun, A. A. Tountas, C. Li, S. Wang, K. Feng, A.-B. Xu, S. Tang, A. Tavasoli, M. Peng, W. Liu, A. S. Helmy, L. He, G. A. Ozin, X. Zhang, "Greenhouse-inspired supra-photothermal CO_2 catalysis" *Nat. Energy* **2021**, *6*, 807–814.

[12] J. Mazarío, S. Ghosh, V. Varela-Izquierdo, L. M. Martínez-Prieto, B. Chaudret, "Magnetic Nanoparticles and Radio Frequency Induction: From Specific Heating to Magnetically Induced Catalysis" *ChemCatChem* **2024**, *0*, e202400683.

[13] T. Len, R. Luque, "Addressing the CO_2 challenge through thermocatalytic hydrogenation to carbon monoxide, methanol and methane" *Green Chem.* **2023**, *25*, 490–521.

[14] J. Artz, T. E. Müller, K. Thenert, J. Kleinekorte, R. Meys, A. Sternberg, A. Bardow, W. Leitner, "Sustainable Conversion of Carbon Dioxide: An Integrated Review of Catalysis and Life Cycle Assessment" *Chem. Rev.* **2018**, *118*, 434–504.

[15] E. T. C. Vogt, B. M. Weckhuysen, "The refinery of the future" *Nature* **2024**, *629*, 295–306.

[16] Y. Zeng, X. Tu, "Plasma-Catalytic CO_2 Hydrogenation at Low Temperatures" *IEEE Trans. Plasma Sci.* **2016**, *44*, 405–411.

[17] J. F. De La Fuente, S. H. Moreno, A. I. Stankiewicz, G. D. Stefanidis, "Reduction of CO_2 with hydrogen in a non-equilibrium microwave plasma reactor" *Int. J. Hydrog. Energy* **2016**, *41*, 21067–21077.

[18] C. Hansen, W. Zhou, E. Brack, Y. Wang, C. Wang, J. Paterson, J. Southouse, C. Copéret, "Decoding the Promotional Effect of Iron in Bimetallic Pt–Fe-nanoparticles on the Low Temperature Reverse Water–Gas Shift Reaction" *J. Am. Chem. Soc.* **2024**, *146*, 27555–27562.

[19] J. Li, D. Wang, W. Xiong, J. Ding, W. Huang, "Interfacial Site Density Engineering of ZnO/Cu Cube Inverse Catalysts for CO_2 Hydrogenation Reactions" *ACS Catal.* **2024**, *14*, 17413–17420.

[20] X. Du, R. Li, H. Xin, Y. Fan, C. Liu, X. Feng, J. Wang, C. Dong, C. Wang, D. Li, Q. Fu, X. Bao, "In-Situ Dynamic Carburization of Mo Oxide with Unprecedented High CO Formation Rate in Reverse Water-Gas Shift Reaction" *Angew. Chem. Int. Ed.* **2024**, *63*, e202411761.

[21] L. Truong-Phuoc, C. Duong-Viet, J.-M. Nhut, A. Pappa, S. Zafeiratos, C. Pham-Huu, "Induction Heating for the Electrification of Catalytic Processes" *ChemSusChem* **2025**, *18*, e202402335.

[22] I. M. Marin, D. D. Masi, L.-M. Lacroix, P.-F. Fazzini, P. W. N. M. van Leeuwen, J. M. Asensio, B. Chaudret, "Hydrodeoxygenation and hydrogenolysis of biomass-based materials using FeNi catalysts and magnetic induction" *Green Chem.* **2021**, *23*, 2025–2036.

[23] H. Kreissl, J. Jin, S.-H. Lin, D. Schüette, S. Störtte, N. Levin, B. Chaudret, A. J. Vorholt, A. Bordet, W. Leitner, "Commercial $Cu_2Cr_2O_5$ Decorated with Iron Carbide Nanoparticles as a Multifunctional Catalyst for Magnetically Induced Continuous-Flow Hydrogenation of Aromatic Ketones" *Angew. Chem. Int. Ed.* **2021**, *60*, 26639–26646.

[24] Y. Liu, N. Cherkasov, P. Gao, J. Fernández, M. R. Lees, E. V. Rebrov, "The enhancement of direct amide synthesis reaction rate over TiO_2@SiO_2@$NiFe_2O_4$ magnetic catalysts in the continuous flow under radiofrequency heating" *J. Catal.* **2017**, *355*, 120–130.

[25] C. Niether, S. Faure, A. Bordet, J. Deseure, M. Chatenet, J. Carrey, B. Chaudret, A. Rouet, "Improved water electrolysis using magnetic heating of FeC–Ni core–shell nanoparticles" *Nat. Energy* **2018**, *3*, 476–483.

[26] S.-H. Lin, W. Hetaba, B. Chaudret, W. Leitner, A. Bordet, "Copper-Decorated Iron Carbide Nanoparticles Heated by Magnetic Induction as Adaptive Multifunctional Catalysts for the Selective Hydrodeoxygenation of Aldehydes" *Adv. Energy Mater.* **2022**, *12*, 2201783.

[27] S.-H. Lin, S. Ahmedi, A. Kretschmer, C. Campalani, Y. Kayser, L. Kang, S. DeBeer, W. Leitner, A. Bordet, "Low pressure amide hydrogenation enabled by magnetocatalysis" *Nat. Commun.* **2025**, *16*, 3464.
[28] A. Bordet, L.-M. Lacroix, K. Soulantica, B. Chaudret, "A New Approach to the Mechanism of Fischer–Tropsch Syntheses Arising from Gas Phase NMR and Mass Spectrometry" *ChemCatChem* **2016**, *8*, 1727–1731.
[29] A. Bordet, L. Lacroix, P. Fazzini, J. Carrey, K. Soulantica, B. Chaudret, "Magnetically Induced Continuous CO_2 Hydrogenation Using Composite Iron Carbide Nanoparticles of Exceptionally High Heating Power" *Angew. Chem.* **2016**, *128*, 16126–16130.
[30] D. De Masi, J. M. Asensio, P. Fazzini, L. Lacroix, B. Chaudret, "Engineering Iron–Nickel Nanoparticles for Magnetically Induced CO_2 Methanation in Continuous Flow" *Angew. Chem.* **2020**, *132*, 6246–6250.
[31] M. G. Vinum, M. R. Almind, J. S. Engbæk, S. B. Vendelbo, M. F. Hansen, C. Frandsen, J. Bendix, P. M. Mortensen, "Dual-Function Cobalt–Nickel Nanoparticles Tailored for High-Temperature Induction-Heated Steam Methane Reforming" *Angew. Chem.* **2018**, *130*, 10729–10733.
[32] L. Truong-Phuoc, A. Essyed, X.-H. Pham, T. Romero, J.-P. Dath, J.-M. Nhut, A. Brazier, L. Vidal, L. Nguyen-Dinh, C. Pham-Huu, "Catalytic methane decomposition process on carbon-based catalyst under contactless induction heating" *Chem. Synth.* **2024**, *4*, 56.
[33] A. García-Zaragoza, J. L. del Río-Rodríguez, C. Cerezo-Navarrete, S. Gutiérrez-Tarriño, M. A. Molina, L. Costley-Wood, J. Mazarío, B. Chaudret, L. M. Martínez-Prieto, A. M. Beale, P. Oña-Burgos, "Pd-Enhanced Carbon-Encapsulated Co Nanoparticles for Efficient Reverse Water–Gas Shift under Magnetic Induction Heating" *ACS Catal.* **2025**, *15*, 9489–9502.
[34] X. Liu, B. Luo, R. Li, J. Hu, C. Dai, X. Ma, C. Song, "Rapid Hydrogen Spillover in Alternating Magnetic Fields Enables Efficient Reduction of CO_2 to CO" *ACS Catal.* **2025**, 10663–10673.
[35] W. Wu, K. Xie, D. Sun, X. Li, F. Fang, "CuO/ZnO/Al_2O_3 Catalyst Prepared by Mechanical-Force-Driven Solid-State Ion Exchange and Its Excellent Catalytic Activity under Internal Cooling Condition" *Ind. Eng. Chem. Res.* **2017**, *56*, 8216–8223.
[36] C. Wang, W. Fang, Z. Liu, L. Wang, Z. Liao, Y. Yang, H. Li, L. Liu, H. Zhou, X. Qin, S. Xu, X. Chu, Y. Wang, A. Zheng, F.-S. Xiao, "Fischer–Tropsch synthesis to olefins boosted by MFI zeolite nanosheets" *Nat. Nanotechnol.* **2022**, *17*, 714–720.
[37] W. Fang, C. Wang, Z. Liu, L. Wang, L. Liu, H. Li, S. Xu, A. Zheng, X. Qin, L. Liu, F.-S. Xiao, "Physical mixing of a catalyst and a hydrophobic polymer promotes CO hydrogenation through dehydration" *Science* **2022**, *377*, 406–410.
[38] C. Wang, J. Du, L. Zeng, Z. Li, Y. Dai, X. Li, Z. Peng, W. Wu, H. Li, J. Zeng, "Direct synthesis of extra-heavy olefins from carbon monoxide and water" *Nat. Commun.* **2023**, *14*, 1857.
[39] A. Bordet, J. M. Asensio, K. Soulantica, B. Chaudret, "Enhancement of Carbon Oxides Hydrogenation on Iron-Based Nanoparticles by In-Situ Water Removal" *ChemCatChem* **2018**, *10*, 4047–4051.
[40] P. Wang, F.-K. Chiang, J. Chai, A. I. Dugulan, J. Dong, W. Chen, R. J. P. Broos, B. Feng, Y. Song, Y. Lv, Q. Lin, R. Wang, I. A. W. Filot, Z. Men, E. J. M. Hensen, "Efficient conversion of syngas to linear α-olefins by phase-pure χ-Fe_5C_2" *Nature* **2024**, *635*, 102–107.
[41] C. Qin, K. Wu, Y. Xu, S. Guo, R. Li, H. Fan, D. Xu, M. Ding, "Accelerated syngas-to-heavy fuels production in heterogeneous catalysis via a proximity effect between a promoter and an active site" *Cell Rep. Phys. Sci.* **2023**, *4*, 101327.
[42] D. De Masi, J. M. Asensio, P. Fazzini, L. Lacroix, B. Chaudret, "Engineering Iron–Nickel Nanoparticles for Magnetically Induced CO_2 Methanation in Continuous Flow" *Angew. Chem. Int. Ed.* **2020**, *59*, 6187–6191.
[43] H. Li, Y. Li, Y. Zhang, C. Liang, H. Wang, B. Li, D. Adair, Z. Bakenov, "Fabrication and Properties of Carbon-Encapsulated Cobalt Nanoparticles over NaCl by CVD" *Nanoscale Res. Lett.* **2016**, *11*, 432.
[44], "FactSage Database Documentation," can be found under https://www.crct.polymtl.ca/fact/documentation/(accessed 10 August 2025), **n.d.**
[45] A. M. Bahmanpour, F. Héroguel, M. Kılıç, C. J. Baranowski, L. Artiglia, U. Röthlisberger, J. S. Luterbacher, O. Kröcher, "Cu–Al Spinel as a Highly Active and Stable Catalyst for the Reverse Water Gas Shift Reaction" *ACS Catal.* **2019**, *9*, 6243–6251.
[46] S. Zafeiratos, G. Ulrich, J.-M. Nhut, C. Michon, C. Pham-Huu, "Electrification of catalytic processes with induction heating: The possible hidden role of non-thermal magnetic fields" *Mater. Today Catal.* **2026**, *12*, 100134.
[47] D. L. J. Broere, I. Čorić, A. Brosnahan, P. L. Holland, "Quantitation of the THF Content in $Fe[N(SiMe_3)_2]_2$·xTHF" Inorg. Chem. **2017**, 56, 3140–3143.
[48] S. Ahmedi, L.-M. Lacroix, D. Demirbas, D. J. SantaLucia, C. Weidenthaler, W. Hetaba, W. Leitner, A. Bordet, "Magnetically Induced Iron-Catalyzed Hydrodeoxygenation of Benzylic Esters and Polyesters" J. Am. Chem. Soc. 2025, 147, 34758–34766.
[49] Y. Shi, Z. Li, Q. Hao, R. Li, Y. Li, L. Guo, S. Ouyang, H. Yuan, T. Zhang, "Hydrophobic Fe-Based Catalyst Derived from Prussian Blue for Enhanced Photothermal Conversion of Syngas to Light Olefins" Adv Funct Materials 2023, 2308670.
